# Lycocasine A, a *Lycopodium* Alkaloid from *Lycopodiastrum casuarinoides* and Its Acid-Sensing Ion Channel 1a Inhibitory Activity

**DOI:** 10.3390/molecules29071581

**Published:** 2024-04-01

**Authors:** Shuai Jiang, Wen-Yan Li, Bei-Bei Gao, Qin-Shi Zhao

**Affiliations:** 1 State Key Laboratory of Phytochemistry and Plant Resources in West China, Kunming Institute of Botany, Chinese Academy of Sciences, Kunming 650201, Chinagaobeibei@mail.kib.ac.cn (B.-B.G.); 2 University of Chinese Academy of Sciences, Beijing 100049, China

**Keywords:** *Lycopodiastrum casuarinoides*, Lycopodiaceae, *Lycopodium* alkaloid, acid-sensing ion channel 1a

## Abstract

A novel *Lycopodium* alkaloid, lycocasine A (**1**), and seven known *Lycopodium* alkaloids (**2**–**8**), were isolated from *Lycopodiastrum casuarinoides*. Their structures were determined through NMR, HRESIMS, and X-ray diffraction analysis. Compound **1** features an unprecedented 5/6/6 tricyclic skeleton, highlighted by a 5-aza-tricyclic[6,3,1,0^2,6^]dodecane motif. In bioactivity assays, compound **1** demonstrated weak inhibitory activity against acid-sensing ion channel 1a.

## 1. Introduction

Rheumatoid arthritis (RA) is an autoimmune disorder characterized by the immune system’s attack on the synovial membrane of joints, leading to inflammation and, in severe cases, permanent damage and disability [1]. Epidemiological studies indicate that around 1% of the global population is afflicted with rheumatoid arthritis. The primary treatment strategy focuses on controlling synovitis, though long-term medication use does not cure the disease and often results in serious side effects [2].

Recent studies showed that acid-sensing ion channel 1a (ASIC1a) was a new therapeutic target for rheumatoid arthritis [2,3]. The ASIC1a, part of the degenerin/epithelial sodium channel family, is activated under acidic conditions and plays a crucial role in managing physiological and pathological states, including inflammation, acidosis, ischemia, and hypoxia [3]. Despite the existence of compounds with notable ASIC1a inhibitory activity, few are derived from plant sources [4], highlighting the significance of exploring plant-based natural products as ASIC1a inhibitors.

Given the long-established connection between rheumatoid arthritis and palindromic rheumatism [5], and the effectiveness of numerous traditional Chinese medicines in treating rheumatism [6], our research aimed at identifying ASIC1a inhibitors from herbal sources for rheumatoid arthritis therapy. *Lycopodiastrum casuarinoides* is a perennial evergreen herb traditionally used for rheumatism treatment [7]. *L. casuarinoides* produces *Lycopodium* alkaloids, the characteristic metabolites of the Lycopodiaceae family [8,9,10], known for their diverse structures and unique skeletons [9,11,12,13,14]. Studies have indicated that *L. casuarinoides* is particularly rich in lycodine-type *Lycopodium* alkaloids [15,16,17,18,19,20], some exhibiting significant anti-cholinesterase [21,22], neuroprotective [23], and cytotoxic activities [21].

**Figure 1:**
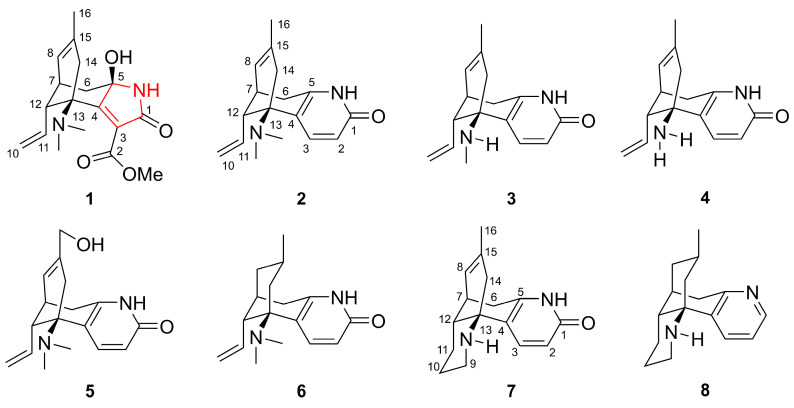
Structures of compounds **1**–**8**.

Our research team has been engaged in the discovery of structurally novel and biologically active *Lycopodium* alkaloids. Previously, we reported fourteen new *Lycopodium* alkaloids from *L. casuarinoides* and their Ca_v_3.1 channel inhibitory activity [24]. Further study on the chemical constituents of *L. casuarinoides* led to the isolation of a new *Lycopodium* alkaloid, lycocasine A (**1**) (Figure 1), and seven known *Lycopodium* alkaloids (**2**–**8**). Lycocasine A (**1**) exhibits a novel 5/6/6 tricyclic skeleton, characterized by a 5-aza-tricyclic[6,3,1,0^2,6^]dodecane motif. Owing to the traditional utilization of *L. casuarinoides* described above, compounds **1**–**8** were evaluated for ASIC1a inhibitory activity and **1** displayed a weak inhibitory effect. This paper elaborates on the isolation, structural elucidation, possible biosynthetic pathway, and ASIC1a inhibitory activity of these compounds.

## 2. Results and Discussion

### 2.1. Structure Elucidation

Lycocasine A (**1**) was isolated as colorless needle-shaped crystals. Its molecular formula was established as C_18_H_24_N_2_O_4_ through HRESIMS *m*/*z* 333.1812 ([M + H]^+^; calculated for C_18_H_25_N_2_O_4_^+^, 333.1809), indicating the presence of eight double-bond equivalents (DBEs). The IR spectrum displayed the presence of one OH/NH (3429 cm^−1^) [25,26], one unsaturated lactam (1694 cm^−1^) [27], and one carbonyl group (1735 cm^−1^) [28]. The ^1^H NMR data (Table 1) revealed an ABX spin system associated with one monosubstituted double bond at *δ*_H_ 6.10 (ddd, *J* = 17.5, 10.7, 7.1 Hz, 1H), 5.15 (br d, *J* = 17.5 Hz, 1H), and 5.11 (br d, *J* = 10.7 Hz, 1H), one vinylic proton at *δ*_H_ 5.48 (br d, *J* = 6.3 Hz, 1H), one vinylic methyl at *δ*_H_ 1.59 (s, 3H), one methoxy group at *δ*_H_ 3.66 (s, 3H), and two *N*-methyls at *δ*_H_ 2.04 (s, 3H) and 2.03 (s, 3H). The ^13^C NMR and DEPT data (Table 1) revealed the presence of eighteen carbon atoms within the molecule, as follows: four methyls (two *N*-methyls at *δ*_C_ 41.5 and 36.2, one methoxy group at *δ*_C_ 51.4), three methylenes (one olefinic at *δ*_C_ 116.5), four methines (two olefinic at *δ*_C_ 138.5 and 124.3), and seven nonprotonated carbons (three olefinic at *δ*_C_ 160.2, 133.4, and 126.9, two carbonyls characteristic of amides or esters at *δ*_C_ 166.0 and 165.3, one carbinolamine moiety at *δ*_C_ 87.0, and one nitrogen-bearing at *δ*_C_ 62.5). The count of six olefinic carbons and two carbonyl groups contributed to five DBEs, suggesting that compound **1** features a tricyclic framework.

**Figure 2:**
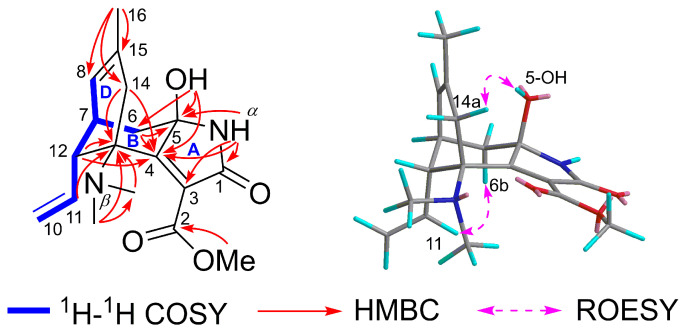
Key 2D NMR correlations of lycocasine A (**1**).

The planar structure of **1** was determined through 2D NMR correlations (Figure 2). The ^1^H−^1^H COSY correlations identified a single spin system as follows: H_2_-10/H-11/H-12/H-7(H-6b)/H-8. The HMBC correlations from *N*-Me(a) to C-13 and *N*-Me(b) and from *N*-Me(b) to C-13 demonstrated their linkage via the *N_β_* atom. The HMBC cross-peaks from H-11 to C-13, from H-12 to C-13 and C-4, from H-6a to C-5 and C-4, and from H-6b to C-5, along with the ^1^H−^1^H COSY correlations of H-12/H-7/H-6b, constructed the ring B. The monosubstituted Δ^10(11)^ double bond was connected to ring B through C-12, as revealed by the ^1^H−^1^H COSY cross-peaks of H_2_-10/H-11/H-12. The ring D was established by the HMBC cross-peaks from H_3_-16 to C-15, C-14, and C-8, and from H_2_-14 to C-13 and C-4, together with the ^1^H−^1^H COSY cross-peaks of H-12/H-7/H-8. The HMBC correlations from 5-OH (*δ*_H_ 5.74) to C-6, C-5, and C-4 and from the *N_α_*H proton (*δ*_H_ 8.76) to C-4 and C-5, along with the chemical shift of C-5 (*δ*_C_ 87.0), fixed 5-OH and *N_α_*H at C-5, which further confirmed the presence of a carbinolamine moiety. Subsequently, the HMBC correlations from the *N_α_*H proton (*δ*_H_ 8.76) to C-1 (*δ*_C_ 166.0), C-3, and C-4 not only indicated the presence of the amide group and Δ^3(4)^ double bond but also constructed the five-membered ring A. The unplaced fragment was attributable to a methyl formate group by the HMBC cross-peak from the OMe (*δ*_H_ 3.66) to C-2 (*δ*_C_ 165.3) and positioned at C-3. Therefore, the planar structure of **1** with a novel 5/6/6 tricyclic skeleton was delineated.

**Figure 3:**
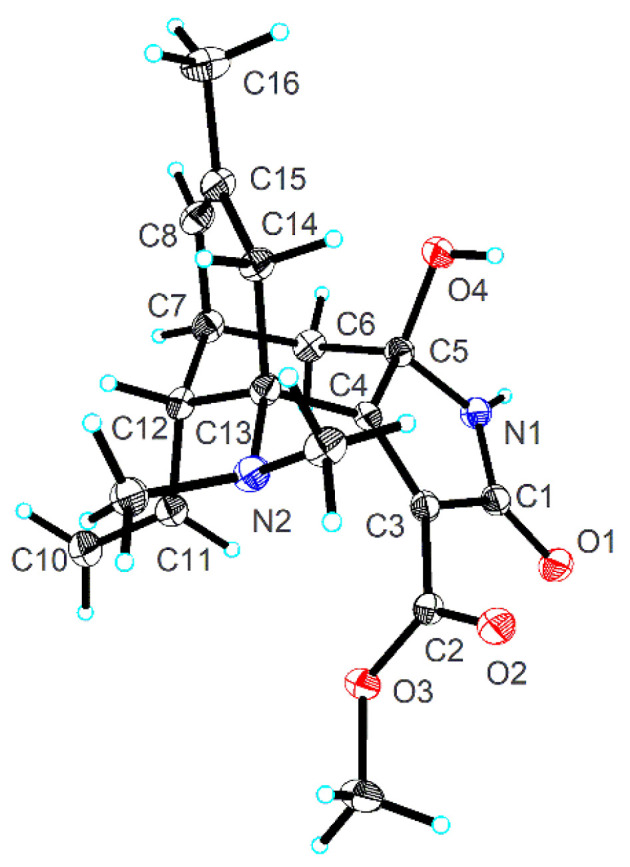
X-ray crystal structure of lycocasine A (**1**).

The relative configuration of **1** was elucidated using ROESY analysis (Figure 2). The correlation of H-11/H-6b indicated the *β*-oriented H-12. The *β*-orientation of 5-OH was defined via the ROESY correlation of 5-OH/H-14a. Single-crystal X-ray diffraction, employing Cu K*α* radiation, conclusively established the structure and determined the absolute configuration as 5*R*,7*R*,12*R*,13*R* [Flack parameter = −0.02(10)] (Figure 3).

**Scheme 1:**
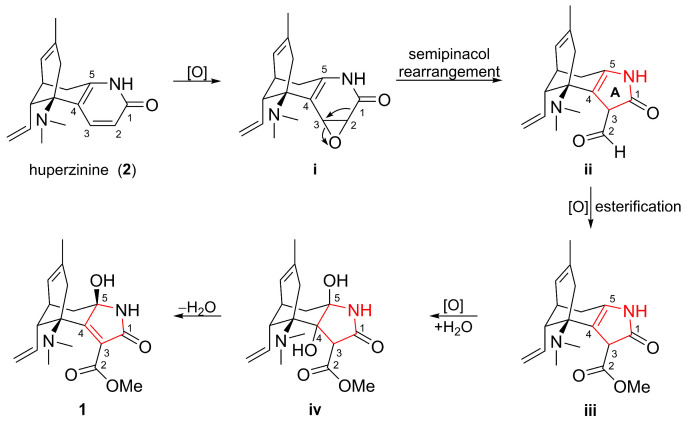
Plausible biosynthetic pathway for **1**.

Biogenetically, compound **1** is hypothesized to originate from huperzinine (**2**) [29,30], a co-isolated, well-known *Lycopodium* alkaloid (Scheme 1). The initial step was the epoxidation [31] of the Δ^2(3)^ double bond of **2** to yield intermediate **i**. Key semipinacol rearrangement [31,32] in **i** led to **ii** with a rearranged five-membered ring A. Finally, oxidation [33] and esterification [33] of the intermediate **ii** was followed by epoxidation [34], epoxide cleavage [34], dehydration [35], and the formation of **1**.

The known *Lycopodium* alkaloids were identified as huperzinine (**2**) [29,30], *N*-demethylhuperzinine (**3**) [30], huperzine C (**4**) [29,36], huperzine D (**5**) [23], 8,15-dihydrohuperzinine (**6**) [37], huperzine B (**7**) [38], and lycodine (**8**) [39] by comparing their 1D NMR data with the literature.

### 2.2. ASIC1a Inhibitory Activity

**Figure 4:**
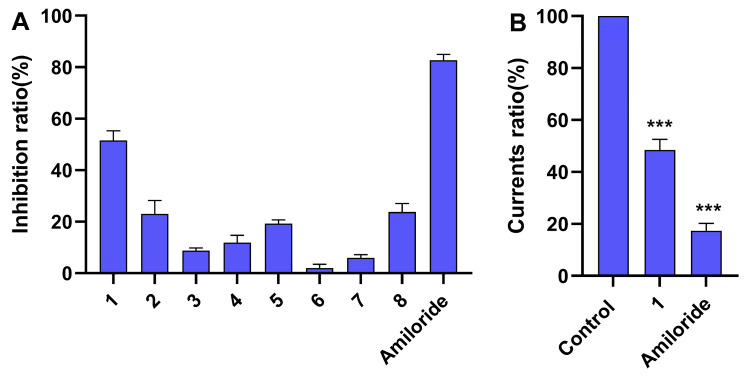
The effects of compounds **1**–**8** and Amiloride on ASIC1a current. (**A**) Inhibition ratio of compounds **1**–**8** (50 µM) and Amiloride (50 µM) on ASIC1a current. The data are presented as the mean ± SD (*n* ≥ 3). (**B**) Current ratio of compound **1** (50 µM) and Amiloride (50 µM) on ASIC1a. The data are presented as the mean ± SD (*n* ≥ 3) and were analyzed by Student’s *t*-test; *** *p* < 0.001 vs. control.

**Figure 5:**
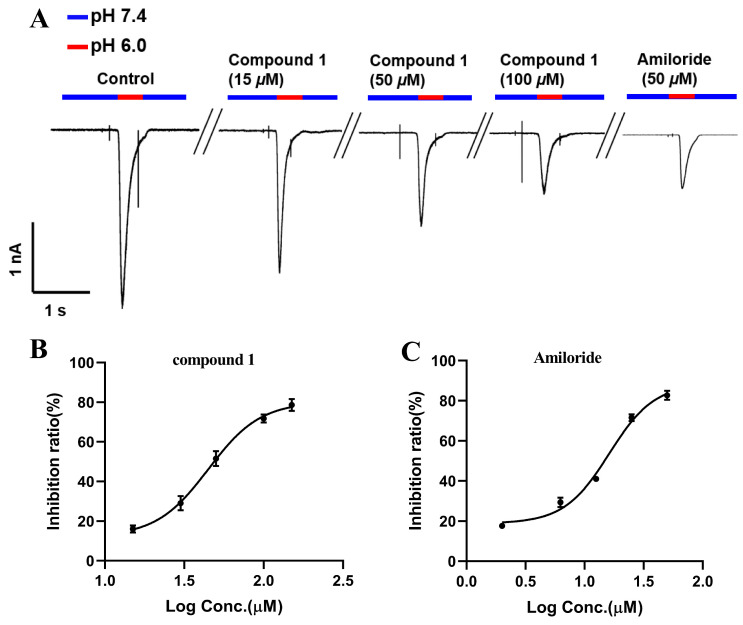
The effects of compound **1** and Amiloride on ASIC1a. (**A**) Illustrative complete-cell ASIC1a currents caused by pH 6.0 with a consistent voltage of −60 mV without (blank control) and with various levels of lycocasine A (**1**) and Amiloride. (**B**) Dose-response of the inhibitory action of lycocasine A (**1**) on the peak current of ASIC1a. The data were fitted using a Hill equation and are presented as the mean ± SD (*n* ≥ 3). (**C**) Dose–response curve of the inhibitory action of Amiloride on the peak current of ASIC1a. The data were fitted using a Hill equation and are presented as the mean ± SD (*n* ≥ 3).

Reflecting the traditional use of *L. casuarinoides*, compounds **1**–**8** were evaluated for their ability to inhibit ASIC1a. Figure 4A shows that compound **1** and Amiloride (positive control) at 50 μM inhibited ASIC1a currents (inhibition rates > 50%). In contrast, compounds **2**–**8** displayed no obvious ASIC1a inhibitory effect (inhibition rates < 30%). Figure 4B displays the statistical analysis of compound **1** (50 µM) and Amiloride (50 µM) on ASIC1a based on the current ratio, indicating their inhibitory effects against ASIC1a (*** *p* < 0.001 vs. control). Figure 5A,B show that compound **1** reduced ASIC1a currents in a concentration-dependent manner, with an IC_50_ value of 48.74 ± 0.92 μM. Amiloride, the inhibitor of ASIC1a, was used as a positive control with an IC_50_ value of 16.15 ± 0.74 μM (Figure 5C). Puerarin, a major compound isolated from the root of *Pueraria lobata*, inhibited ASIC1a with an IC_50_ value of 9.31 µM [40]. Lindoldhamine, a plant bisbenzylisoquinoline alkaloid from the leaves of *Laurus nobilis*, inhibited the ASIC1a with an IC_50_ value of 9 µM [41]. Sinomenine, a plant alkaloid from the roots of *Sinomenium acutum*, inhibits ASIC1a (IC_50_~1 µM) [42]. Compared with Amiloride and these inhibitors of vegetal origin, lycocasine A (**1**) showed a weak inhibitory effect on ASIC1a.

### 2.3. Molecular Docking

**Figure 6:**
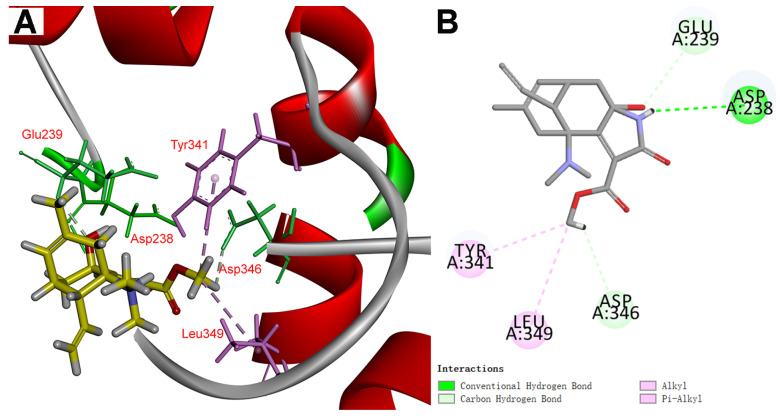
Molecular docking of lycocasine A (**1**) with the cASIC1 protein (PDB code: 6X9H). (**A**) 3D diagram of the interactions of lycocasine A (**1**) with cASIC1 protein. (**B**) 2D diagram of the interactions of lycocasine A (**1**) with the cASIC1 protein.

The interactions of lycocasine A (**1**) with the ASIC1a were revealed by the molecular docking. Given the lack of mammalian ASIC1a co-crystallized with potent small-molecule inhibitors, a chicken ASIC1 protein [43] (cASIC1 protein, PDB code: 6X9H, a homolog of mammalian ASIC1a protein) was selected as the receptor protein for molecular docking. In Figure 6A,B, the docking result shows one pi-alkyl interaction of OMe carbon atom with TYR341, an amino acid with which JNJ-799760 interacts in the crystal of the cASIC1/JNJ-799760 complex, as reported by Michael Maher’s research team [43]. Additionally, hydrogen bond interactions with ASP238, GLU239, and ASP346 and an alkyl interaction with LEU349 were also observed. Moreover, the LibDockscore (45.60) and binding energy (−35.62 Kcal/mol) in the active site indicates the weak interactions between lycocasine A (**1**) and cASIC1 protein. These results may elucidate the weak inhibitory effect of lycocasine A (**1**) on ASIC1a.

## 3. Materials and Methods

### 3.1. General Experimental Procedures

The melting point was identified utilizing a WRX-4 micro melting point machine (Shanghai Yice Apparatus & Equipments Co. Ltd., Shanghai, China). UV spectra were characterized using a Chirascan V100 spectrophotometer (Applied Photophysics Ltd., Leatherhead, Surrey, UK). Optical activity was characterized using a Rudolph Autopol VI polarimeter (Rudolph Research Analytical, Hackettstown, NJ, USA). IR spectra were acquired with a Bruker VERTEX 70 spectrometer (Bruker, Karlsruhe, Germany) using KBr pellets, while NMR data were obtained using Bruker AV-400 MHz apparatuse (Bruker, Karlsruhe, Germany), Bruker AV-600 MHz apparatuse (Bruker, Karlsruhe, Germany) or Bruker AV-800 MHz apparatuse (Bruker, Karlsruhe, Germany) with 5 mm probe. The chemical shift values were outlined utilizing the residual DMSO-*d_6_* (*δ*_H_ 2.50 ppm), chloroform-*d_1_* (*δ*_H_ 7.26 ppm), or methanol-*d_4_* (*δ*_H_ 3.30 ppm) as an internal standard for the ^1^H NMR spectrometry and DMSO-*d_6_* (*δ*_C_ 39.5 ppm), chloroform-*d_1_* (*δ*_C_ 77.0 ppm), or methanol-*d_4_* (*δ*_C_ 49.0 ppm) for the ^13^C NMR spectrometry (see Supplementary Materials (Tables S1–S8) for detailed NMR parameters). HRESIMS were acquired on an Agilent 1290 UPLC/6540 Q-TOF mass spectrometer (Agilent Technologies, Palo Alto, CA, USA). Preparative HPLC was performed with an Agilent 1260 (Agilent Technologies, Palo Alto, CA, USA) using a XBridge-C18 column (5 μm, 1 × 25 cm, Waters Corporation, Milford, MA, USA). The column chromatography employed a C18-CE column (40 μm, 5 × 31 cm, Zhejiang Acchrom Technology Co., Ltd., Wenling, China) or silica gel column (200–300 mesh). The TLC plates (GF_254_) were obtained from Haixiang Chemical Co., Ltd. (Linyi, China). The alkaloids on the TLC plates were detected by spraying the plates with Dragendorff reagent.

### 3.2. Plant Material

*L. casuarinoides* was obtained from Fujian Province, China, in March 2021. It was authenticated by Prof. Xiao Cheng (the Kunming Institute of Botany, KIB). The specific sample (No. 202103009) was stored in the Kunming Institute of Botany.

### 3.3. Extraction and Isolation

All parts of the *L. casuarinoides* (80 kg) were powdered and percolated with methanol at ambient temperature for six days (90 L × 3). Following solvent removal under reduced pressure, the obtained sample was separated using 1‰ H_2_SO_4_ and EtOAc. The H_2_O residue was modified to pH 9.0 using 1‰ NaOH and extracted using trichloromethane to give a crude total alkaloid (45 g). This fraction underwent silica gel column chromatography, eluted with gradients of petroleum ether/acetone (8:2–0:10) and acetone/methanol (9:1–0:10), resulting in three portions (Fr.A to Fr.C). Fr.A (19.8 g) underwent recrystallization to yield compound **2** (18 g). The mother solution was partitioned by a C18-CE column (methanol/H_2_O/NH_3_·H_2_O, 1:9:0.005 to 10:0:0.005) to afford three subfractions (Fr.A.1 to Fr.A.3). Compound **8** (53 mg) was obtained from Fr.A.1 through the silica gel column employing petroleum ether/acetone (6:4) as the eluent. Fr.A.2 was purified by the silica gel column using trichloromethane/methanol (9.7:0.3 to 0:10) to obtain compound **6** (8 mg). Fr.A.3 was isolated by a silica gel column (EtOAc/methanol, 9.7:0.3 to 0:10) and purified via HPLC eluting using methanol/H_2_O/NH_3_·H_2_O (4.5:5.5:0.005, 3.6 mL/min) to yield compound **1** (1.6 mg, *t*_R_ = 20.5 min). Fr.B (4.3 g) underwent a C18-CE column (methanol/H_2_O/NH_3_·H_2_O, 1:9:0.005 to 10:0:0.005) and recrystallization to afford compound **3** (2.9 g). Fr. C (12.8 g) was chromatographed over a C18-CE column (methanol/H_2_O/NH_3_·H_2_O, 1:9:0.005 to 10:0:0.005), resulting in the isolation of three fractions (Fr.C.1 to Fr.C.3). Fr.C.1 underwent recrystallization to furnish compound **4** (0.9 g). Fr.C.2 was chromatographed using a silica gel column (trichloromethane/methanol, 9.5:0.5 to 0:10) to give compound **7** (93 mg). Compound **5** (18 mg) was isolated from Fr.C.3 by a silica gel column (EtOAc/methanol, 9.5:0.5 to 0:10).

#### Lycocasine A (**1**)

For the lycocasine A (**1**), the following apply: colorless needle-shaped crystals; mp 187–189 °C; [*α*${]}_{D}^{19}$ +51.0 (*c* 0.16, MeOH); IR (KBr) *ν*_max_ 3429, 2947, 2925, 1735, 1694, 1648, 1635, 1436, 1384, 1102, and 1077 cm^−1^; ^1^H and ^13^C NMR data, see Table 1; and positive HRESIMS *m*/*z* 333.1812 [M + H]^+^ (calculated for C_18_H_25_N_2_O_4_^+^, 333.1809).

### 3.4. Crystallographic Data of Lycocasine A (**1**)

The colorless needle-shaped crystals of compound **1** were obtained from methanol by slow evaporation at room temperature. X-ray crystallography was conducted with a diffractometer (Bruker APEX DUO, Bruker, Karlsruhe, Germany) alongside Cu K*α* radiation. The data from compound **1** were placed in the Cambridge Crystallographic Data Center (CCDC 2330436). The data are freely available at https://www.ccdc.cam.ac.uk/structures/? (accessed on 7 February 2024) (or from the Cambridge Crystallographic Data Center, 12 Union Road, Cambridge CB2 1EZ, UK; Fax: (+44)-1223-336033; e-mail: data_request@ccdc.cam.ac.uk).

The crystal data for **1** were as follows: C_18_H_24_N_2_O_4_, *M* = 332.39, *a* = 7.4849(2) Å, *b* = 26.1713(7) Å, *c* = 13.4238(3) Å, *α* = 90°, *β* = 93.1000(10)°, *γ* = 90°, *V* = 2625.73(12) Å^3^, *T* = 150.(2) K, space group *P*1211, *Z* = 6, μ(Cu Kα) = 0.730 mm^−1^, 39,840 metrical reflexions, and 9897 individual reflexions (*R_int_* = 0.0975). The final values of *R_1_* and *wR*(*F*^2^) were 0.0392 and 0.0868 (*I* > 2*σ*(*I*)), respectively. The final values of *R_1_* and *wR*(*F*^2^) were 0.0483 and 0.0924 (all data), respectively. The goodness of fit of *F*^2^ was 1.036. The value of the flack parameter was −0.02(10).

### 3.5. Cell Transfection and Electrophysiological Recordings

HEK293T cells, acquired from ATCC, were cultured at 37 °C in a 5% CO_2_ atmosphere using Dulbecco’s Modified Eagle Medium (DMEM, Gibco, Thermo Fisher Scientific, Inc., Waltham, MA, USA) supplemented with glucose, L-glutamine, pyruvate, 10% fetal bovine serum (FBS, VivaCell, Shanghai VivaCell Biosciences Ltd., Shanghai, China), and 1% penicillin–streptomycin (Pen-Strep, VivaCell, Shanghai VivaCell Biosciences Ltd, Shanghai, China). Cells were plated at a low density in 12-well plates 24 h prior to transfection. For transfection, Lipofectamine 3000 (Invitrogen, Thermo Fisher Scientific, Inc., Waltham, MA, USA) was utilized to introduce 300 ng of ASIC1a cDNA into adherent cells, which were then analyzed for 24–48 h post-transfection. Complete-cell voltage-clamp measurements were obtained at ambient temperature (24 °C), holding the cell membrane voltage at −60 mV. The ASIC1a greatest currents were triggered by a solution pH of 6.0, keeping the holding potential at −60 mV. Borosilicate glass micropipettes, fashioned to achieve a resistance of 2–6 MΩ, were filled with an intracellular recording solution comprising 140 mM KCl, 2 mM MgCl_2_, 5 mM EGTA, 5 mM NaCl, and 10 mM HEPES (adjusted to pH 7.4 with KOH). The extracellular recording solution consisted of 145 mM NaCl, 5 mM KCl, 1 mM MgCl_2_, 2 mM CaCl_2_, and 10 mM HEPES (pH 7.4 with NaOH) or 10 mM MES (pH 6.0 with HCl). Amiloride (MedChemExpress, Monmouth Junction, NJ, USA) was used as a positive control. Current signals were amplified using a SUTTER IPA-2 amplifier, with data acquisition and analysis performed using SutterPatch 9.0 software. Data processing was carried out using GraphPad Prism version 8.0.

### 3.6. Molecular Docking

Molecular docking was performed using Discovery Studio 4.0 software. The crystal structure of chicken ASIC1 protein [43] (cASIC1 protein, PDB code: 6X9H, a homolog of mammalian ASIC1a protein) was obtained from the Protein Data Bank. After removing the water molecules, hydrogens and charges were added to the system. The docking site was defined based on the position of the co-crystallized inhibitor. The 3D conformations of compound **1** were optimized by Discovery Studio 4.0 and the top scoring ligand poses were saved. Docking analysis was performed by the LibDock protocol and the docking parameters were set as defaults. The docking results with the highest LibDock score were visualized and are presented in the full text [44].

## 4. Conclusions

In summary, we have identified a novel *Lycopodium* alkaloid, lycocasine A (**1**), and seven known *Lycopodium* alkaloids (**2**–**8**) from *L. casuarinoides*. Compound **1** is characterized by an unprecedented 5/6/6 tricyclic skeleton with a 5-aza-tricyclic[6,3,1,0^2,6^]dodecane moiety. In bioactivity assays, compound **1** exhibited weak inhibition of the ASIC1a. These results not only extend the chemical diversity of *Lycopodium* alkaloid but also provide a solid basis for further exploration of *Lycopodium* alkaloids as ASIC1a inhibitors in the treatment of rheumatoid arthritis.

## Figures and Tables

**Table 1:** ^1^H (800 MHz) and ^13^C (200 MHz) NMR data of compound **1** (*δ*_H_ in ppm, *J* in Hz).

No.	1	
	*δ*_H_	*δ*_C_
1		166.0
2		165.3
3		126.9
4		160.2
5		87.0
6a	2.03, br d (13.7)	40.7
6b	1.54, br dd (13.7, 5.2)	
7	2.31, m	38.7
8	5.48, br d (6.3)	124.3
10a	5.15, br d (17.5)	116.5
10b	5.11, br d (10.7)	
11	6.10, ddd (17.5, 10.7, 7.1)	138.5
12	2.66, br d (7.1)	49.1
13		62.5
14a	2.10, d (17.9)	30.6
14b	2.06, d (17.9)	
15		133.4
16	1.59, s	22.2
*N*-Me(a)	2.04, s	41.5
*N*-Me(b)	2.03, s	36.2
OMe	3.66, s	51.4
NH	8.76, s	
OH	5.74, br s	

## Data Availability

Data are contained within the article or Supplementary Materials.

## Supplementary Materials

The following supporting information can be downloaded at: https://www.mdpi.com/article/10.3390/molecules29071581/s1: Figures S1–S10: 1D and 2D NMR, UV, IR, HREIMS, and HPLC spectra of compound **1**; Figures S11–S24: 1D NMR spectra of compounds **2**–**8**; Tables S1–S8: Detailed NMR parameters of compounds **1**–**8**.
