# Supplementary material for: Lycocasine A, a Lycopodium Alkaloid from Lycopodiastrum casuarinoides and Its Acid-Sensing Ion Channel 1a Inhibitory Activity
Source: Molecules. 2024 Apr 1;29(7):1581. doi: 10.3390/molecules29071581 (PMC11013477; doi:10.3390/molecules29071581)
Supplement: Supplementary file 1 [file molecules-29-01581-s001.zip › molecules-2887740-supplementary.pdf]

Lycocasine A, A *Lycopodium* Alkaloid from *Lycopodiastrum casuarinoides* and Its Acid-Sensing Ion Channel 1a Inhibitory Activity

Shuai Jiang [1,2,†], Wen-Yan Li [1,2,†], Bei-Bei Gao [1,2] and Qin-Shi Zhao [1,2,*]

[1] State Key Laboratory of Phytochemistry and Plant Resources in West China, Kunming Institute of Botany, Chinese Academy of Sciences, Kunming 650201, Yunnan, China
[2] University of Chinese Academy of Sciences, Beijing 100049, China
* Correspondence: qinshizhao@mail.kib.ac.cn (Q.-S.Z.); Tel.: +86-871-65223058
† These authors contributed equally to this work.

Contents of Supplementary Information

Figure S1. ^{1}H NMR spectrum of compound **1** in DMSO-d_6 (800 MHz) 3
Figure S2. ^{13}C NMR and DEPT spectra of compound **1** in DMSO-d_6 (200 MHz) 3
Figure S3. HSQC spectrum of compound **1** in DMSO-d_6 (800 MHz) 4
Figure S4. HMBC spectrum of compound **1** in DMSO-d_6 (800 MHz) 4
Figure S5. ^{1}H–^{1}H COSY spectrum of compound **1** in DMSO-d_6 (800 MHz) 5
Figure S6. ROESY spectrum of compound **1** in DMSO-d_6 (800 MHz) 5
Figure S7. UV spectrum of compound **1** 6
Figure S8. IR spectrum of compound **1** 6
Figure S9. HRESIMS spectrum of compound **1** 7
Figure S10. HPLC chromatogram of compound **1** (MeOH/H_2O/$NH_3 \cdot H_2O$, 45:55:0.05, 3.6 mL/min) 7
Figure S11. ^{1}H NMR spectrum of compound **2** in $CDCl_3$ (400 MHz) 8
Figure S12. ^{13}C NMR and DEPT spectra of compound **2** in $CDCl_3$ (100 MHz) 8
Figure S13. ^{1}H NMR spectrum of compound **3** in $CDCl_3$ (400 MHz) 9
Figure S14. ^{13}C NMR and DEPT spectra of compound **3** in $CDCl_3$ (100 MHz) 9
Figure S15. ^{1}H NMR spectrum of compound **4** in $CDCl_3$ (400 MHz) 10
Figure S16. ^{13}C NMR and DEPT spectra of compound **4** in $CDCl_3$ (100 MHz) 10
Figure S17. ^{1}H NMR spectrum of compound **5** in $CDCl_3$ (600 MHz) 11
Figure S18. ^{13}C NMR and DEPT spectra of compound **5** in $CDCl_3$ (150 MHz) 11
Figure S19. ^{1}H NMR spectrum of compound **6** in $CDCl_3$ (600 MHz) 12
Figure S20. ^{13}C NMR and DEPT spectra of compound **6** in $CDCl_3$ (150 MHz) 12
Figure S21. ^{1}H NMR spectrum of compound **7** in CD_3OD (400 MHz) 13
Figure S22. ^{13}C NMR and DEPT spectra of compound **7** in CD_3OD (100 MHz) 13
Figure S23. ^{1}H NMR spectrum of compound **8** in $CDCl_3$ (600 MHz) 14
Figure S24. ^{13}C NMR and DEPT spectra of compound **8** in $CDCl_3$ (150 MHz) 14

Table S1. Detailed NMR parameters of compound **1**· 15
Table S2. Detailed NMR parameters of compound **2**· 15
Table S3. Detailed NMR parameters of compound **3**· 16
Table S4. Detailed NMR parameters of compound **4**· 16
Table S5. Detailed NMR parameters of compound **5**· 16
Table S6. Detailed NMR parameters of compound **6**· 17
Table S7. Detailed NMR parameters of compound **7**· 17
Table S8. Detailed NMR parameters of compound **8**· 17

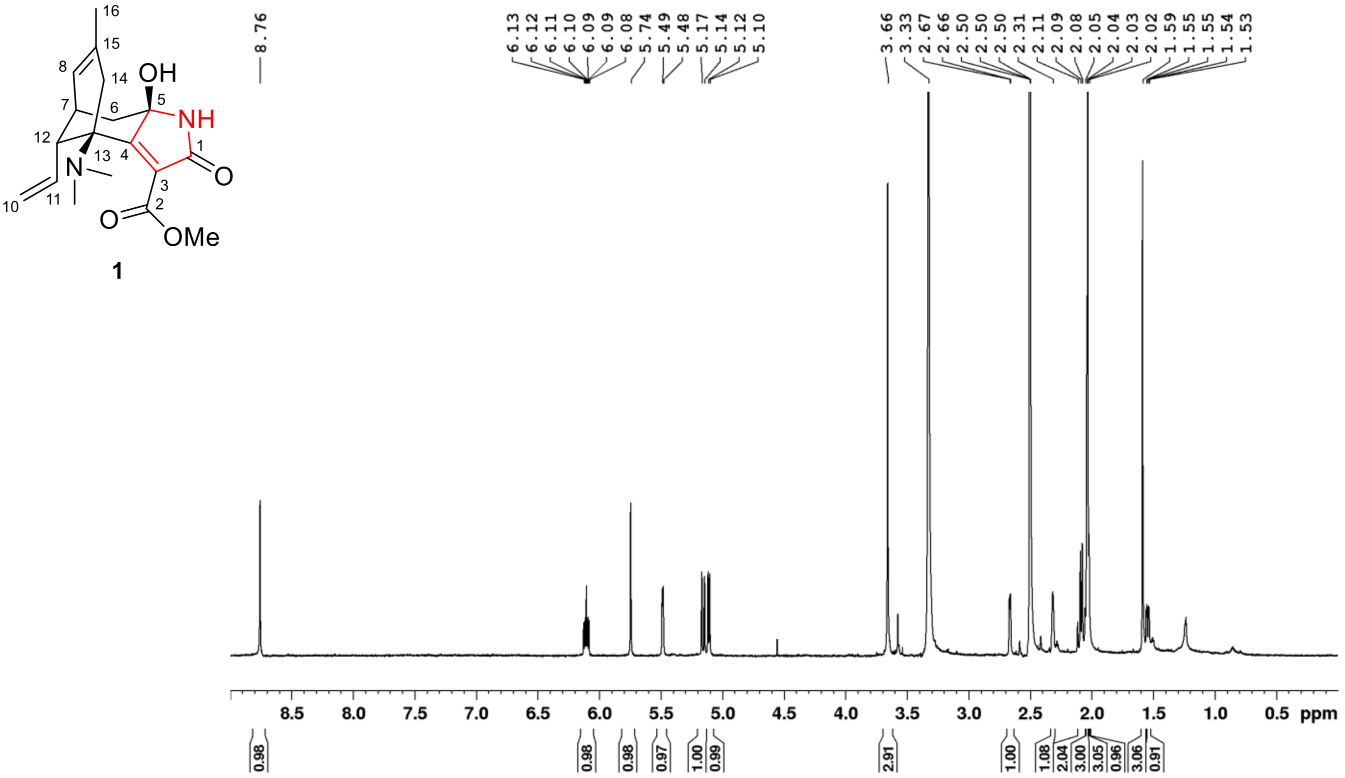

Figure S1. ^{1}H NMR spectrum of compound **1** in DMSO-d_6 (800 MHz)

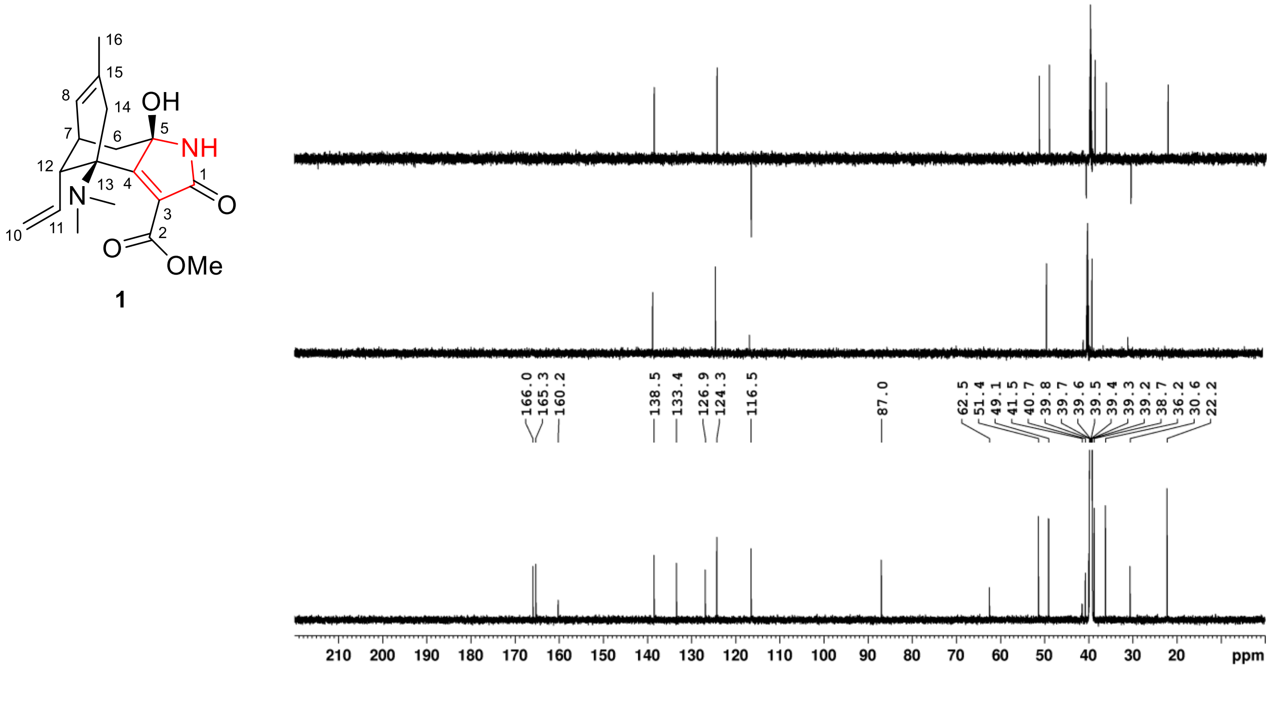

Figure S2. ^{13}C NMR and DEPT spectra of compound **1** in DMSO-d_6 (200 MHz)

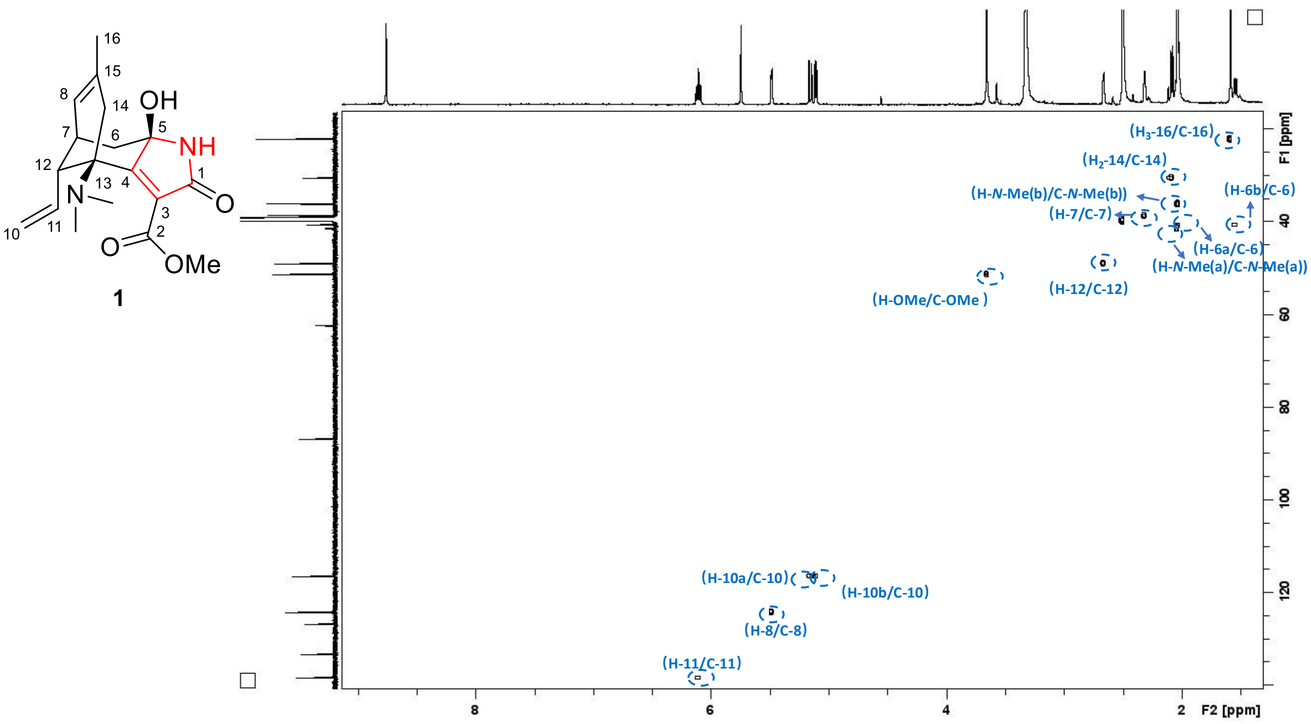

Figure S3. HSQC spectrum of compound **1** in DMSO-d_6 (800 MHz)

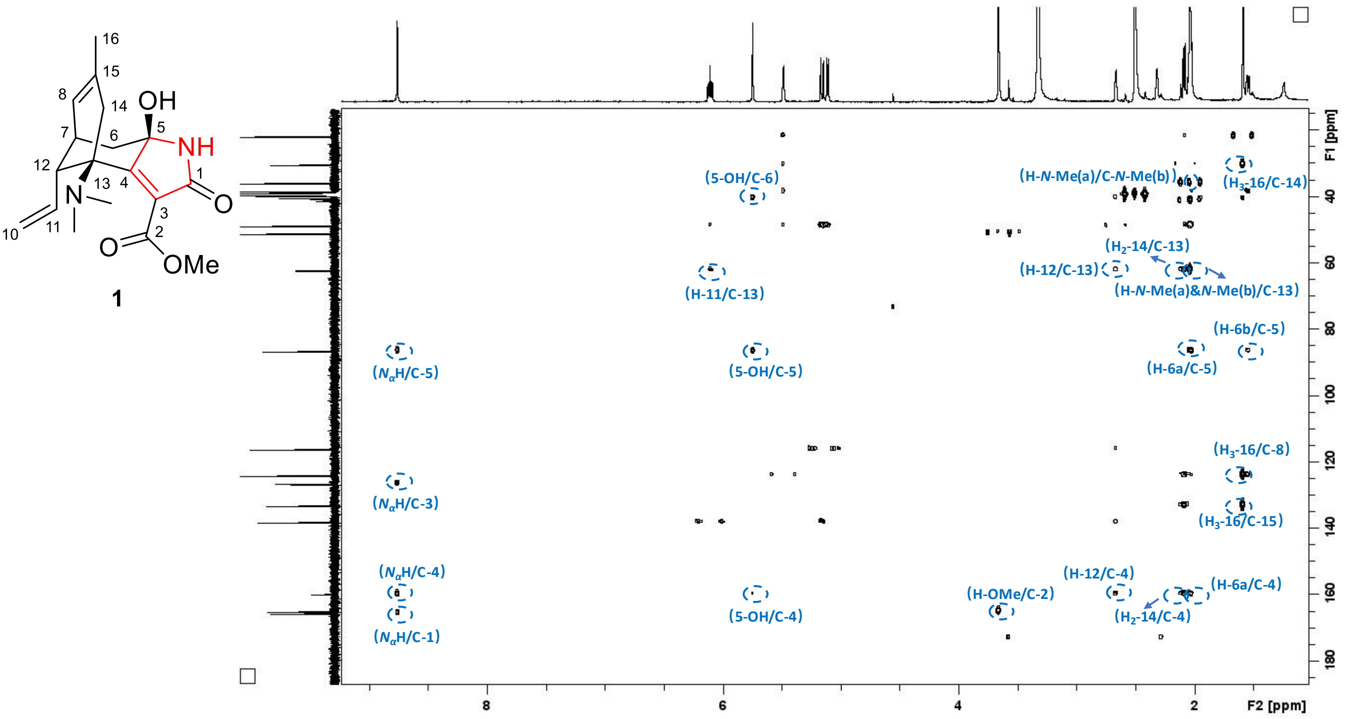

Figure S4. HMBC spectrum of compound **1** in DMSO-d_6 (800 MHz)

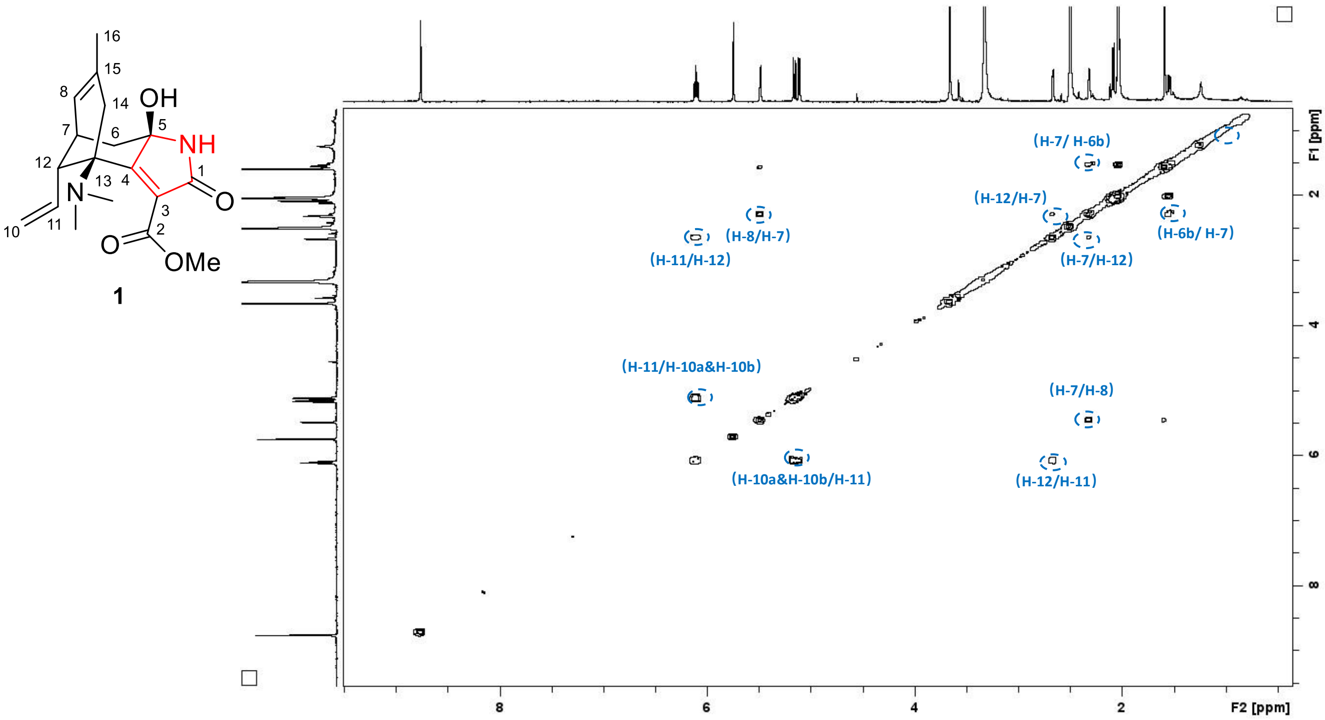

Figure S5. $^{1}H–^{1}H$ COSY spectrum of compound **1** in DMSO-d_6 (800 MHz)

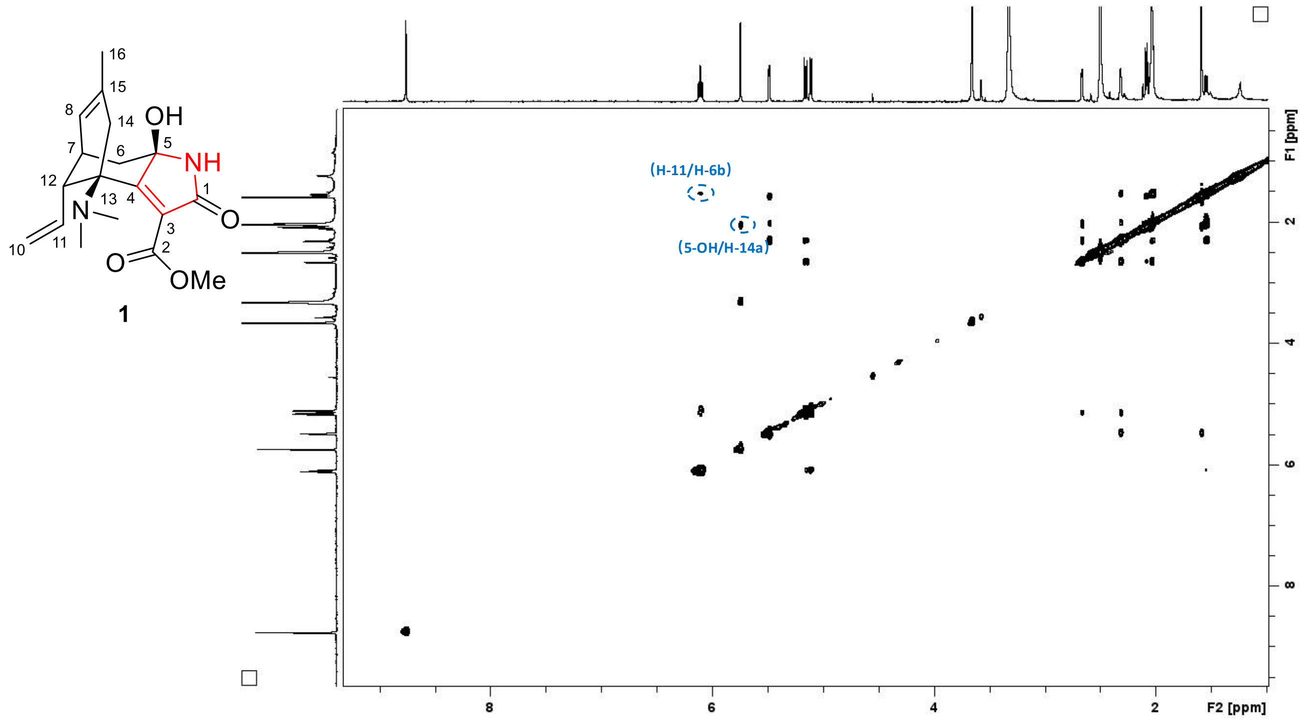

Figure S6. ROESY spectrum of compound **1** in DMSO-d_6 (800 MHz)

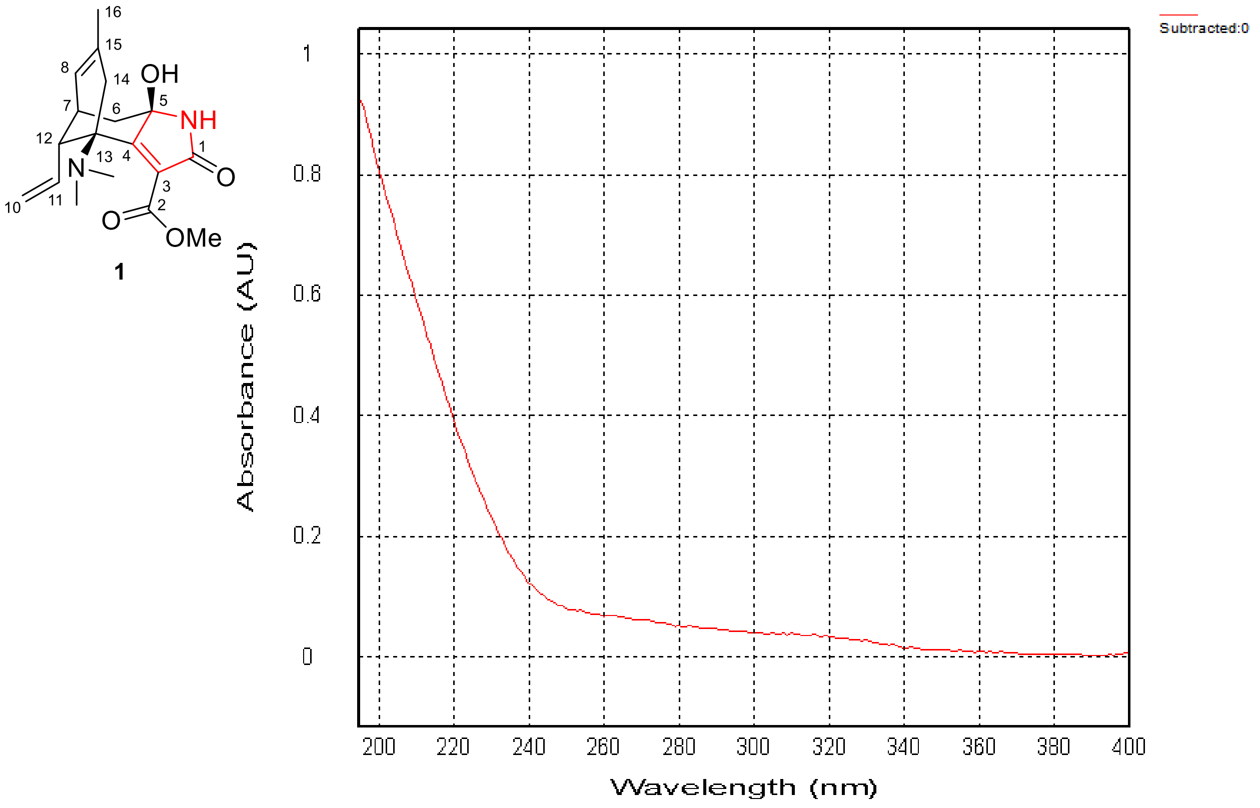

Figure S7. UV spectrum of compound **1**

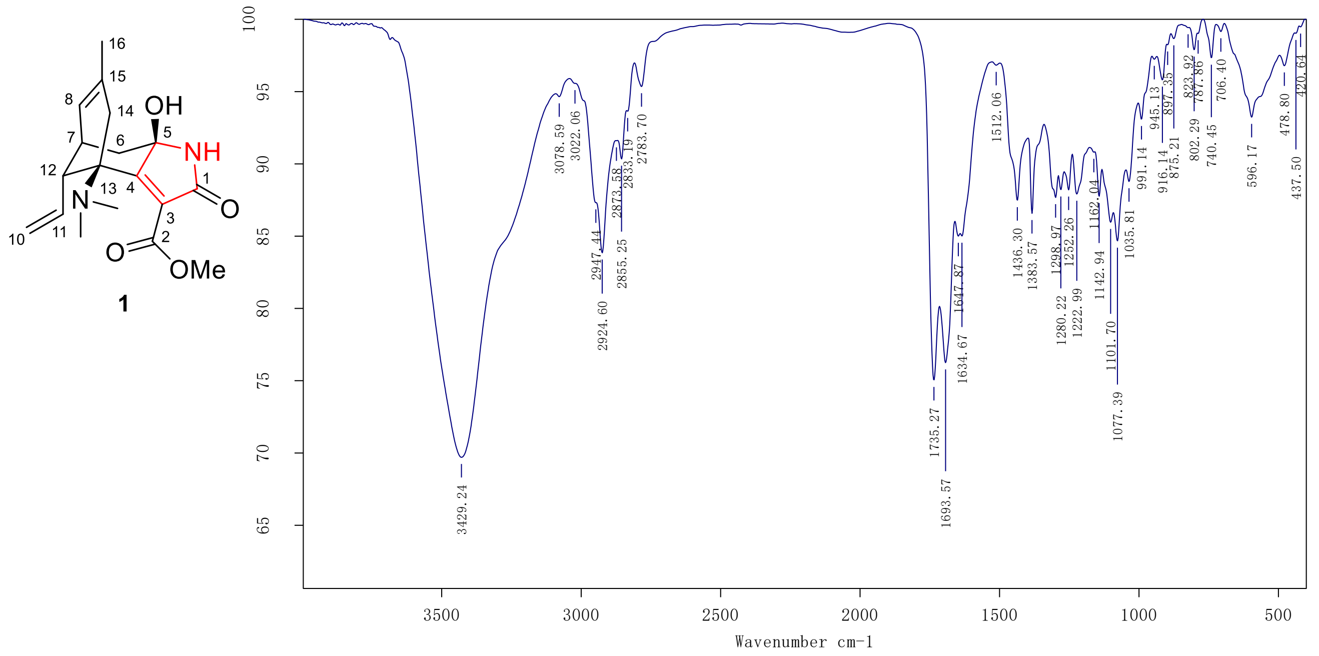

Figure S8. IR spectrum of compound **1**

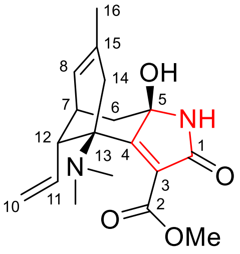

1

User Spectra

Fragmentor Voltage	Collision Energy	Ionization Mode
135	0	ESI

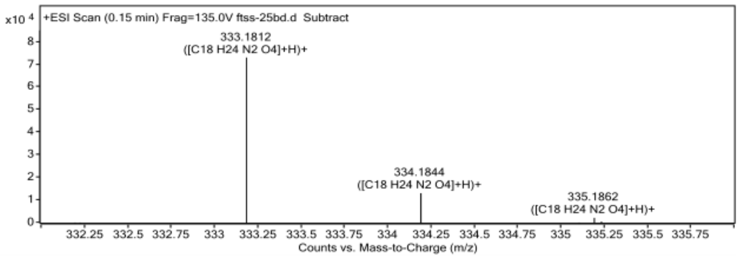

Peak List

m/z	z	Abund	Formula	Ion
333.1812	1	73158.77	C18 H24 N2 O4	(M+H)+
334.1844	1	13353.29	C18 H24 N2 O4	(M+H)+
355.1632	1	61820.29		
356.1664	1	9945.4		
357.2172	1	7649.1		
371.1365	1	5903.56		
415.1517	1	8126.96		
687.3372	1	55840.3		
688.3398	1	21394.15		
689.3424	1	5442.51		

Formula Calculator Element Limits

Element	Min	Max
C	3	60
H	0	120
O	0	30
N	0	5

Formula Calculator Results

Formula	CalculatedMass	CalculatedMz	Mz	Diff. (mDa)	Diff. (ppm)	DBE
C18 H24 N2 O4	332.1736	333.1809	333.1812	-0.30	-0.90	8.0000

--- End Of Report ---

Figure S9. HRESIMS spectrum of compound **1**

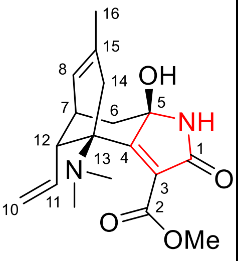

1

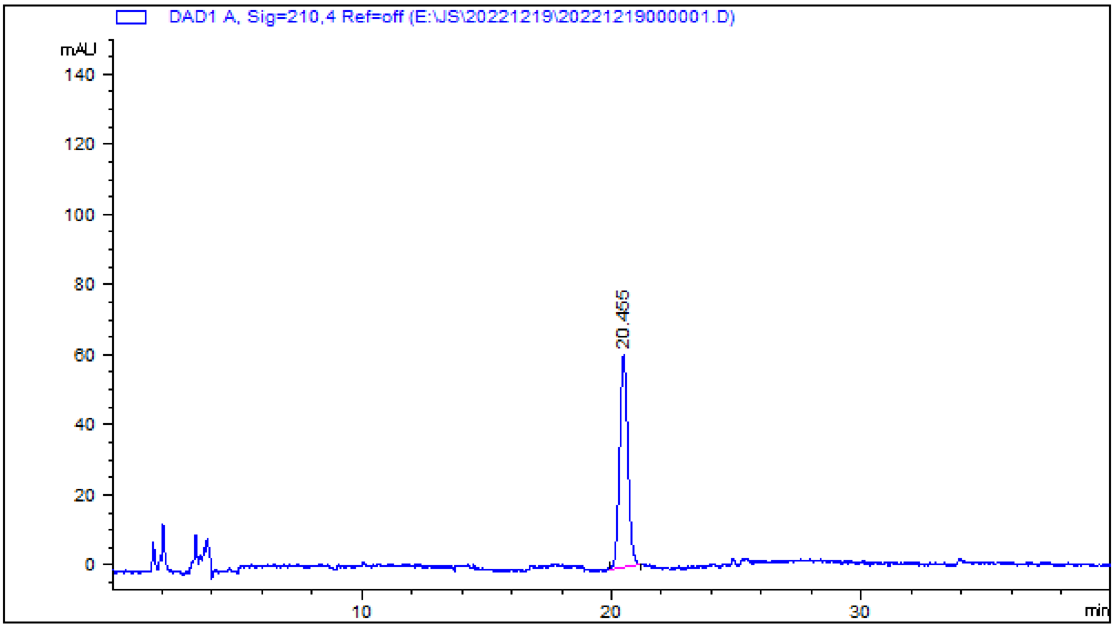

Figure S10. HPLC chromatogram of compound **1** (MeOH/H_2O/$NH_3\cdot H_2O$, 4.5:5.5:0.005, 3.6 mL/min)

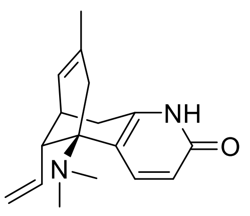

2

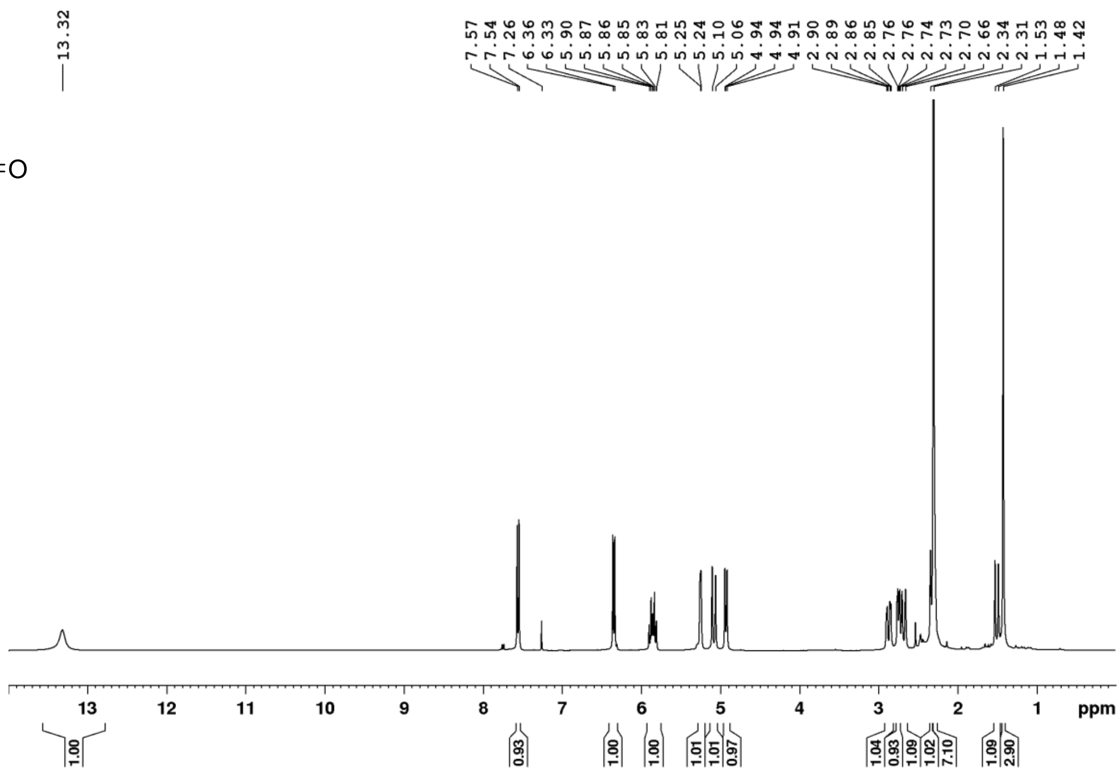

Figure S11. ^{1}H NMR spectrum of compound **2** in $CDCl_3$ (400 MHz)

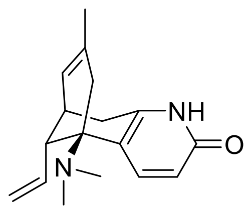

2

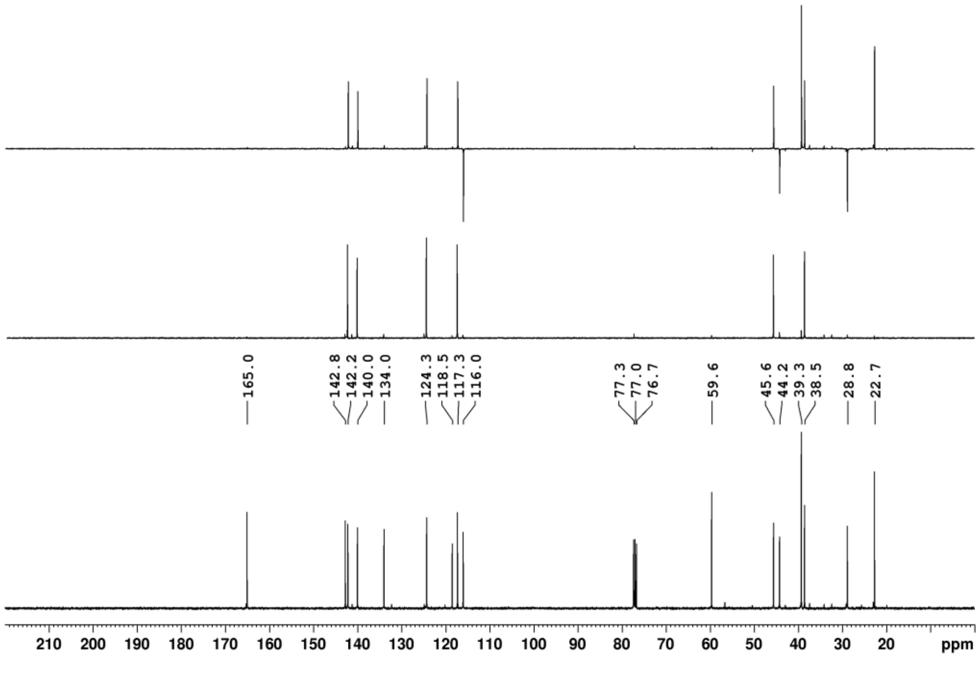

Figure S12. ^{13}C NMR and DEPT spectra of compound **2** in $CDCl_3$ (100 MHz)

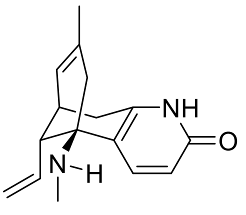

3

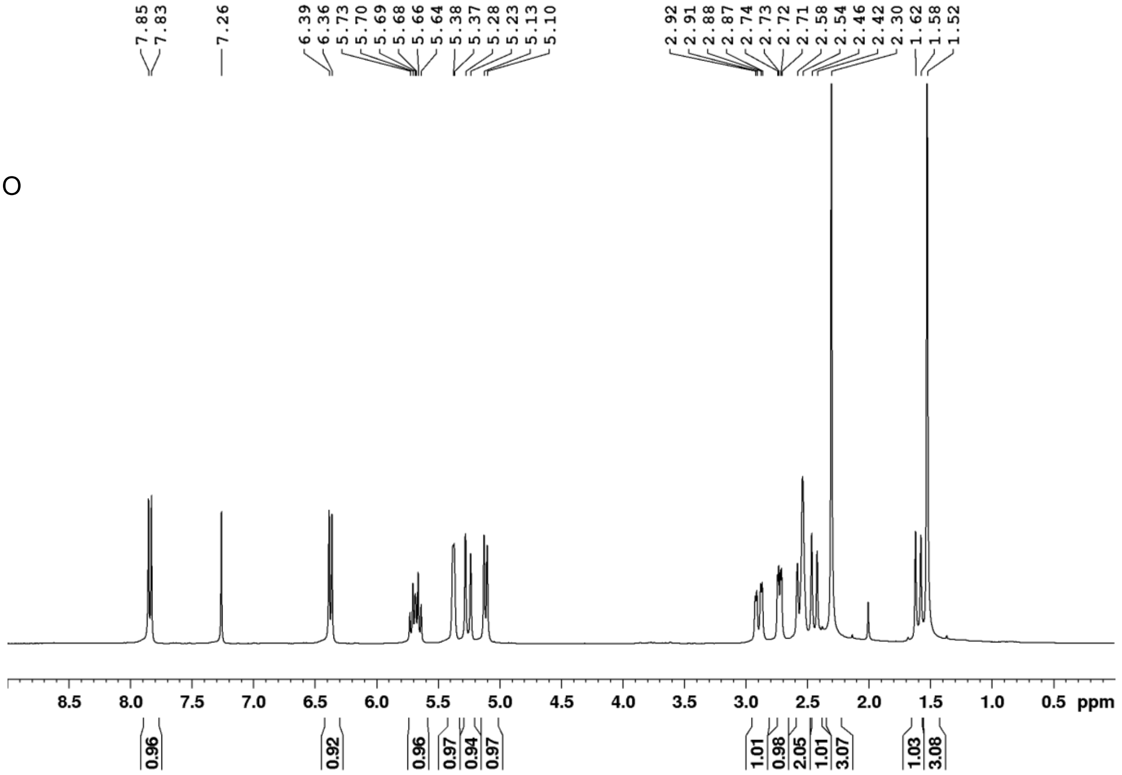

Figure S13. ^{1}H NMR spectrum of compound **3** in $CDCl_3$ (400 MHz)

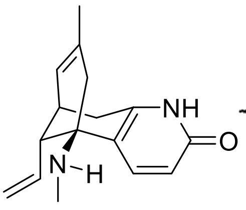

3

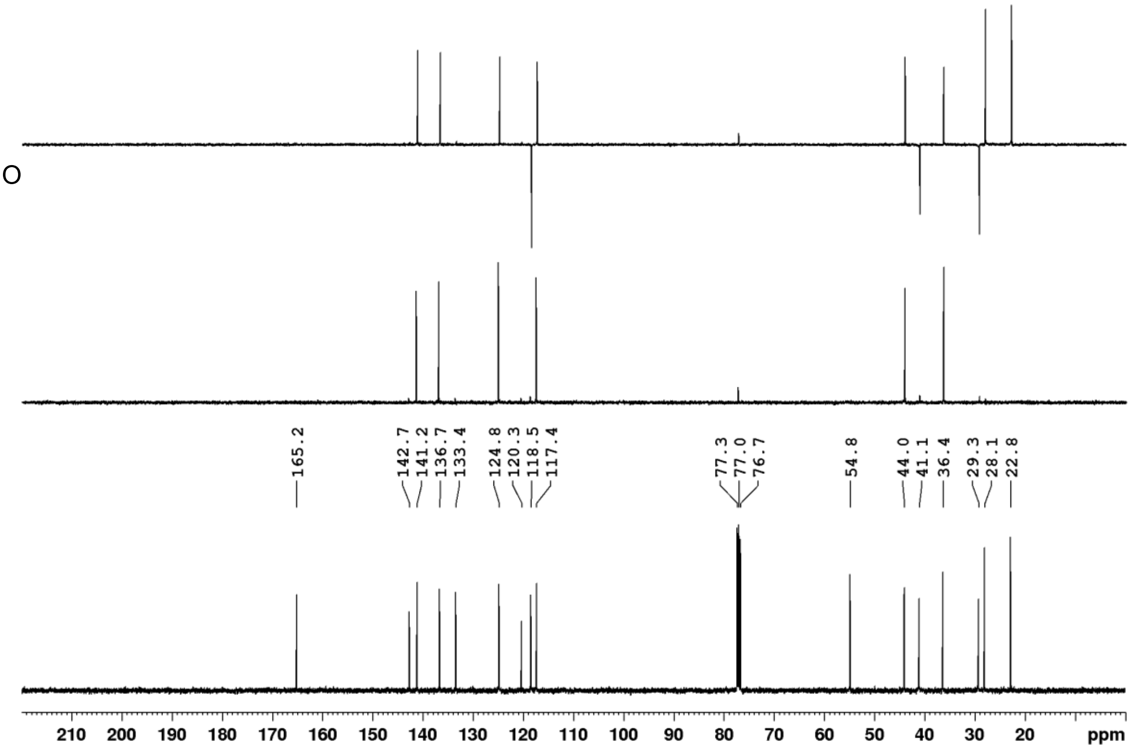

Figure S14. ^{13}C NMR and DEPT spectra of compound **3** in $CDCl_3$ (100 MHz)

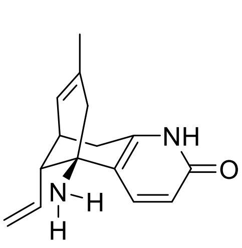

4

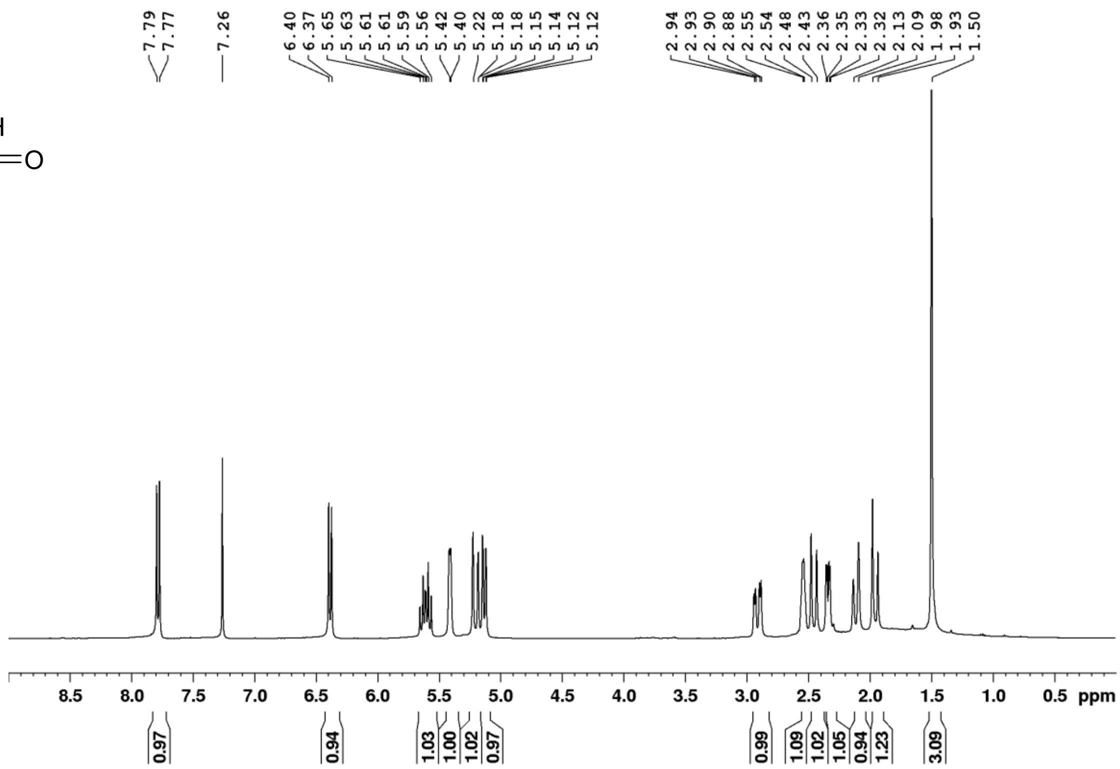

Figure S15. ^{1}H NMR spectrum of compound **4** in $CDCl_3$ (400 MHz)

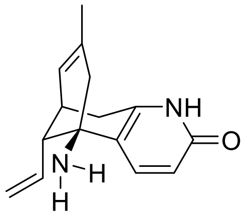

4

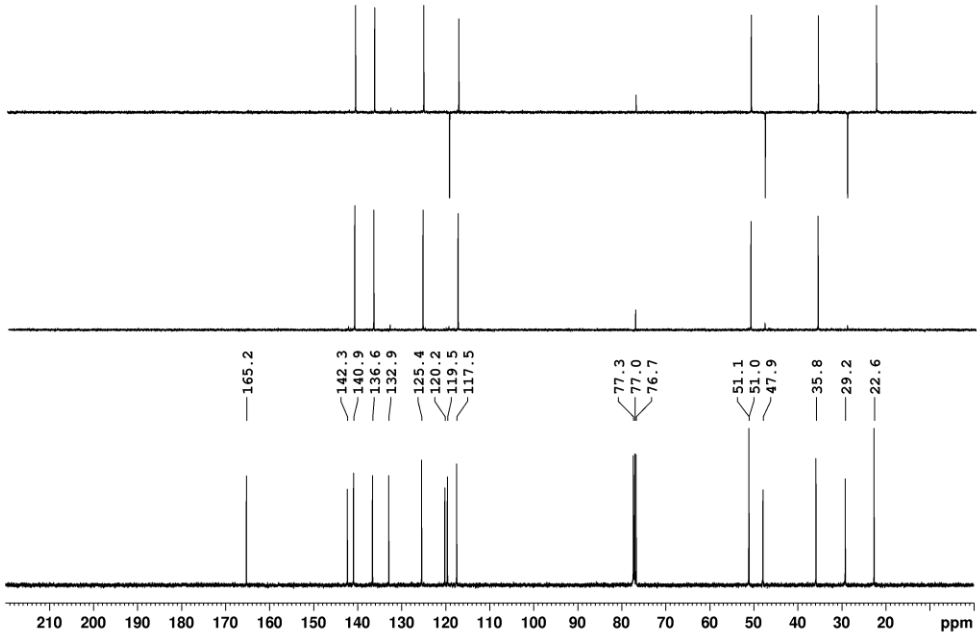

Figure S16. ^{13}C NMR and DEPT spectra of compound **4** in $CDCl_3$ (100 MHz)

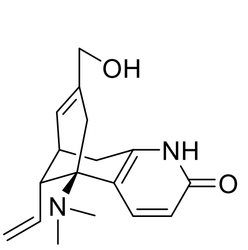

5

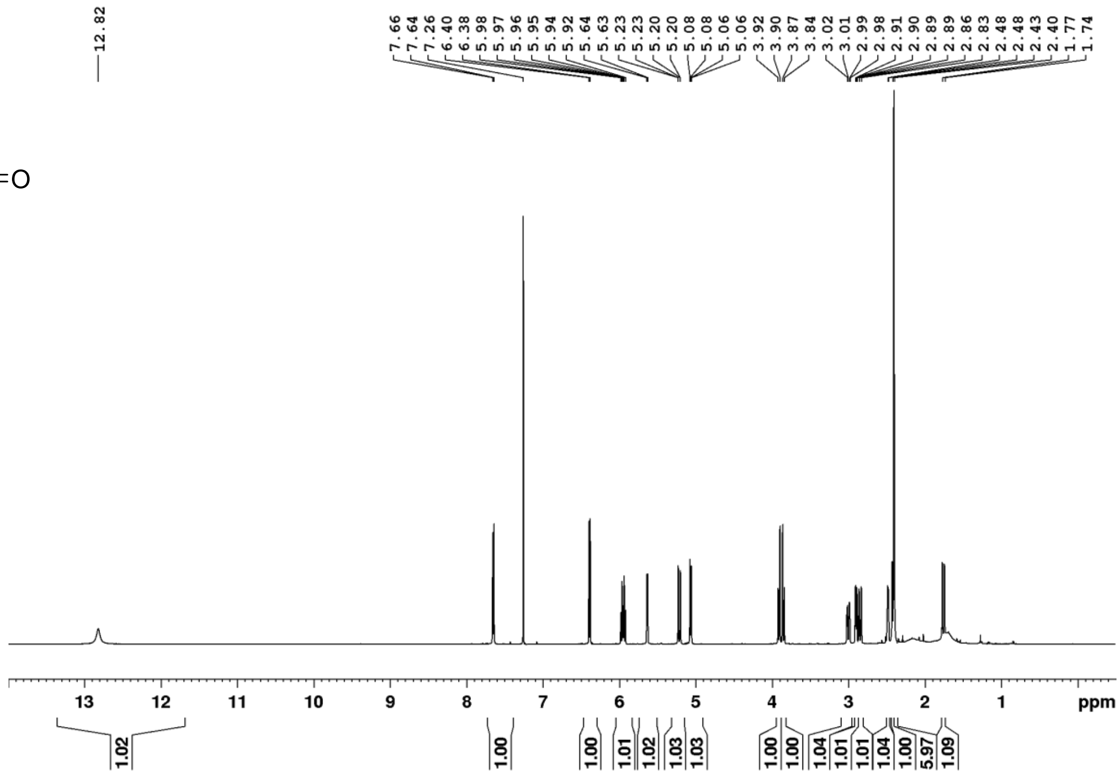

Figure S17. ^{1}H NMR spectrum of compound **5** in $CDCl_3$ (600 MHz)

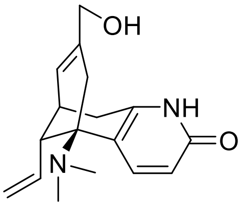

5

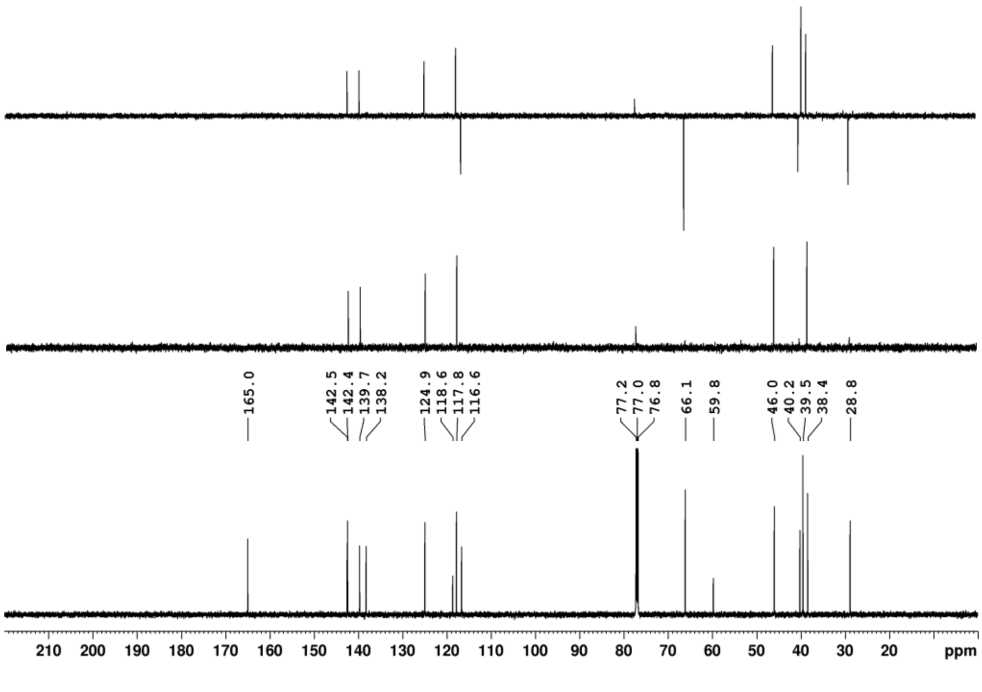

Figure S18. ^{13}C NMR and DEPT spectra of compound **5** in $CDCl_3$ (150 MHz)

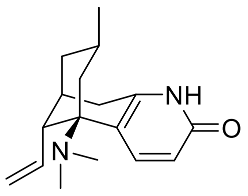

6

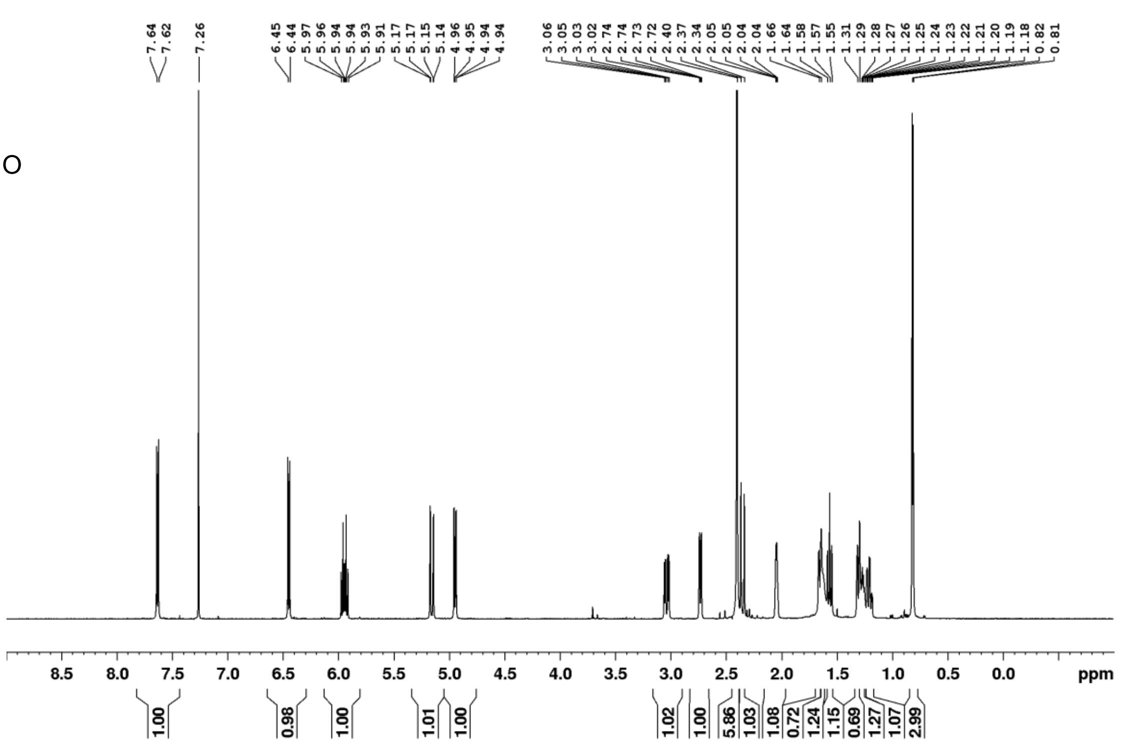

Figure S19. ^{1}H NMR spectrum of compound **6** in $CDCl_3$ (600 MHz)

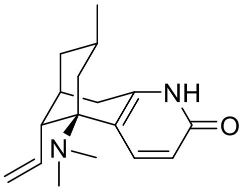

6

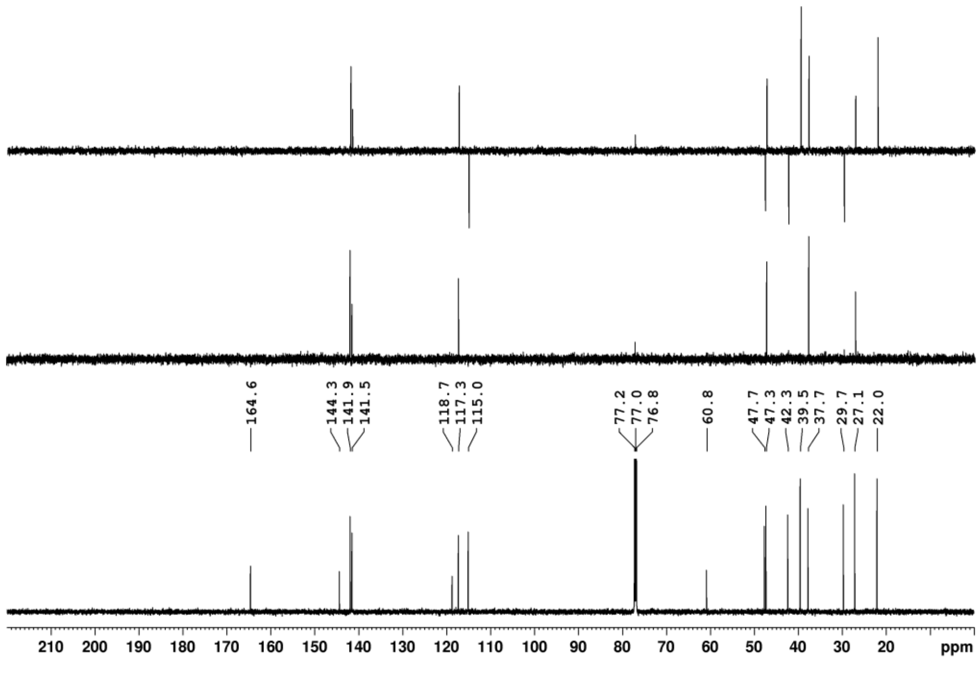

Figure S20. ^{13}C NMR and DEPT spectra of compound **6** in $CDCl_3$ (150 MHz)

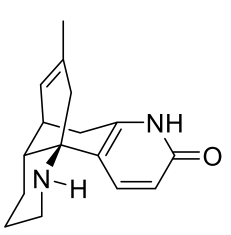

7

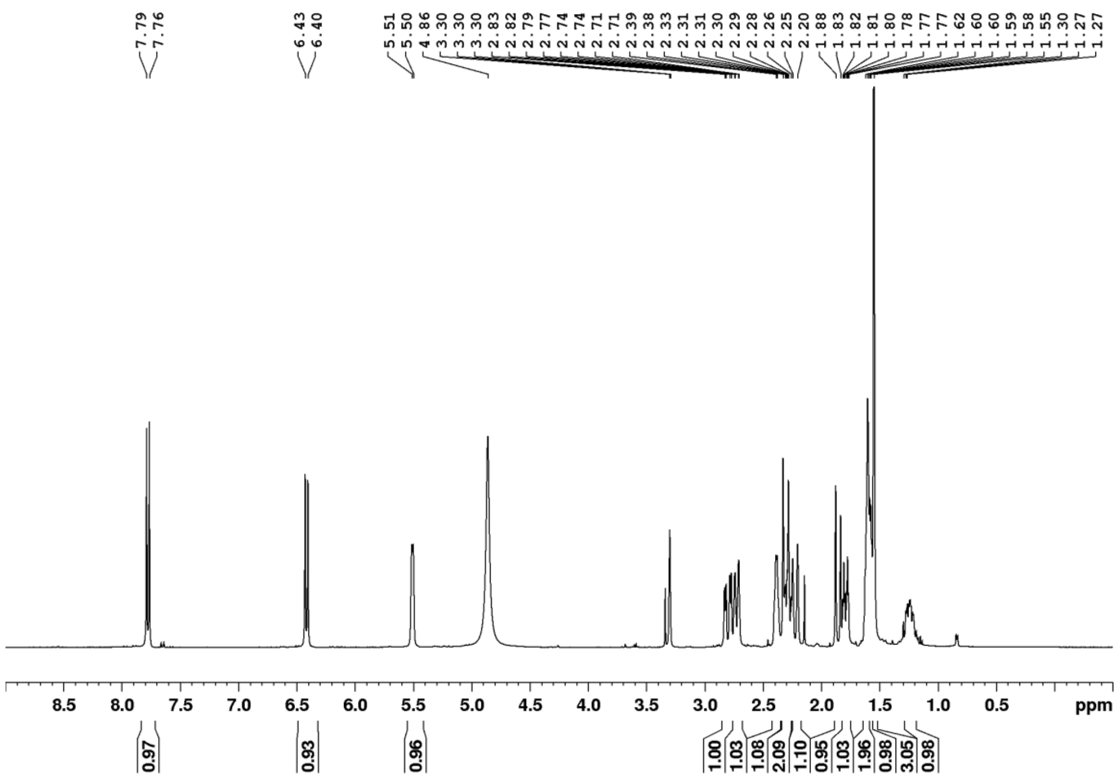

Figure S21. ^{1}H NMR spectrum of compound **7** in CD_3OD (400 MHz)

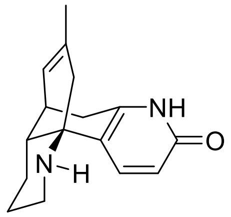

7

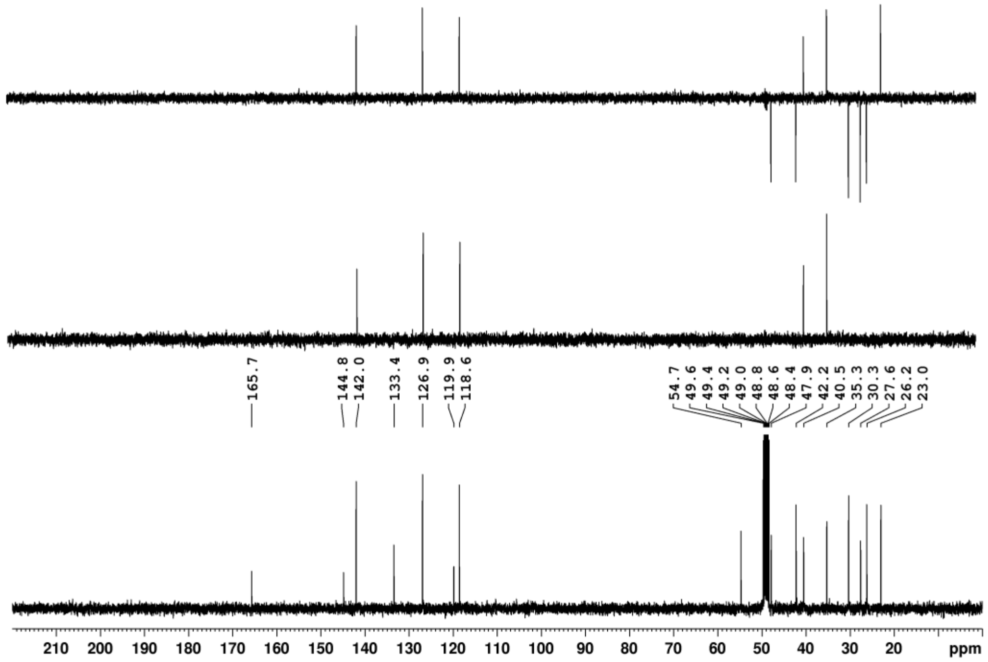

Figure S22. ^{13}C NMR and DEPT spectra of compound **7** in CD_3OD (100 MHz)

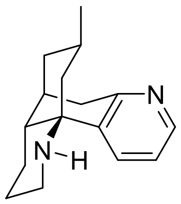

8

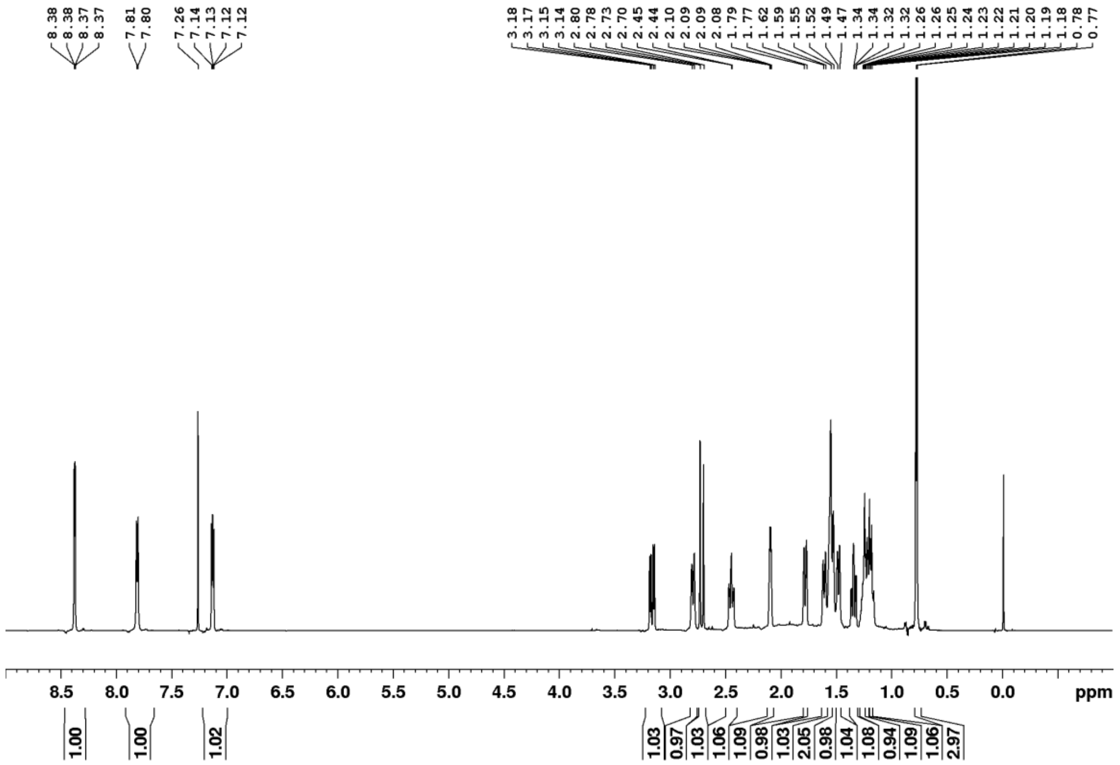

Figure S23. ^{1}H NMR spectrum of compound **8** in $CDCl_3$ (600 MHz)

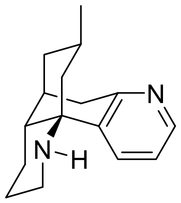

8

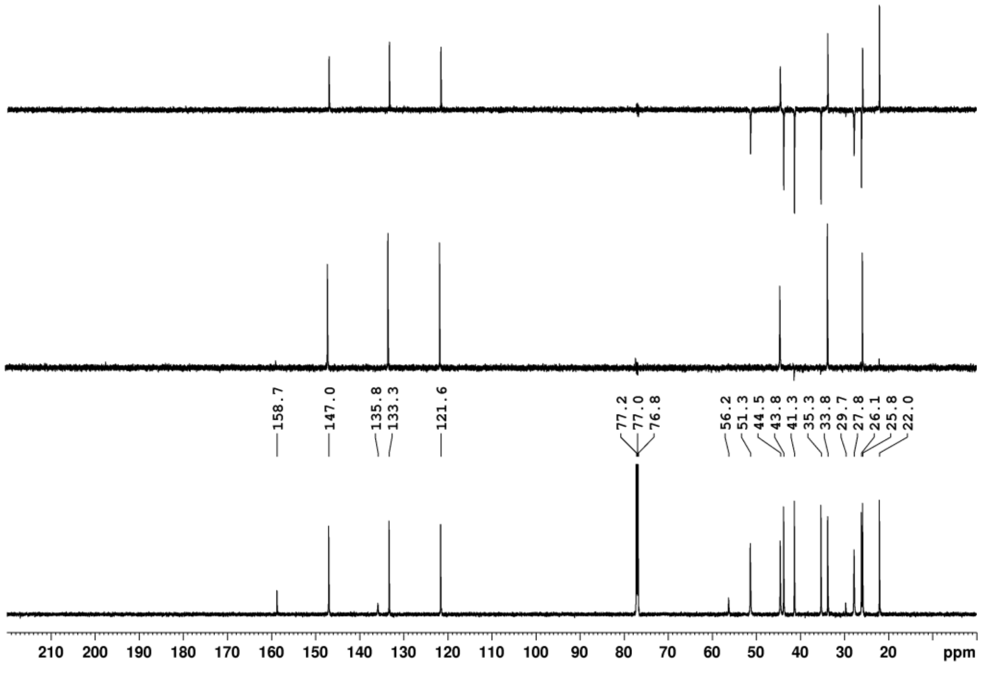

Figure S24. ^{13}C NMR and DEPT spectra of compound **8** in $CDCl_3$ (150 MHz)

spectra	^{1}H		^{13}C		DEPT90		DEPT135	
pulse sequence	zg30		zgdc		deptsp90		deptsp135	
temperature [k]	300.0		300.0		300.0		300.0	
relaxation delay (D1) time [sec]	2		2		2		2	
number of scans (NS)	4		1000		300		300	
frequency [MHz]	800		200		200		200	
sweep width (SW) [Hz]	16025		48076		48076		48076	
acquisition time (AQ) [sec]	2.044		0.681		0.681		0.681	
spectra	HSQC		HMBC		^{1}H–^{1}H COSY		ROESY	
pulse sequence	hsqcetgpprsisp2.2		hmbcgpndqf		cosygpppqf		roesygpph19	
temperature [k]	300.0		300.0		300.0		300.0	
relaxation delay (D1) time [sec]	1.5		1.5		1		1.5	
number of scans (NS)	8		24		6		16	
f	f2	f1	f2	f1	f2	f1	f2	f2
frequency [MHz]	800	200	800	200	800	800	800	800
sweep width (SW) [Hz]	12019	30188	8802	45082	12019	12019	12019	12019
acquisition time (AQ) [sec]	0.085	0.002	0.232	0.232	0.085	0.007	0.085	0.085

Table S1. Detailed NMR parameters of compound **1**.

spectra	^{1}H	^{13}C	DEPT90	DEPT135
pulse sequence	zg	zgpg30	dept90	dept135
temperature [k]	300.0	300.0	300.0	300.0
relaxation delay (D1) time [sec]	2	2	2	2
number of scans (NS)	4	70	35	70
frequency [MHz]	400	100	100	100
sweep width (SW) [Hz]	8012	24038	24038	23809
acquisition time (AQ) [sec]	4.089	1.363	1.363	1.376

Table S2. Detailed NMR parameters of compound **2**.

spectra	^{1}H	^{13}C	DEPT90	DEPT135
pulse sequence	zg	zgpg30	dept90	dept135
temperature [k]	300.0	300.0	300.0	300.0
relaxation delay (D1) time [sec]	2	2	2	2
number of scans (NS)	4	70	35	70
frequency [MHz]	400	100	100	100
sweep width (SW) [Hz]	8012	24038	24038	23809
acquisition time (AQ) [sec]	4.089	1.363	1.363	1.376

Table S3. Detailed NMR parameters of compound **3**.

spectra	^{1}H	^{13}C	DEPT90	DEPT135
pulse sequence	zg	zgpg30	dept90	dept135
temperature [k]	300.0	300.0	300.0	300.0
relaxation delay (D1) time [sec]	2	2	2	2
number of scans (NS)	4	85	40	45
frequency [MHz]	400	100	100	100
sweep width (SW) [Hz]	8012	24038	24038	23809
acquisition time (AQ) [sec]	4.089	1.363	1.363	1.376

Table S4. Detailed NMR parameters of compound **4**.

spectra	^{1}H	^{13}C	DEPT90	DEPT135
pulse sequence	zg30	zgdc	dept90	dept135
temperature [k]	300.0	300.0	300.0	300.0
relaxation delay (D1) time [sec]	1	2	2	2
number of scans (NS)	4	256	64	128
frequency [MHz]	600	150	150	150
sweep width (SW) [Hz]	16741	39062	39062	46875
acquisition time (AQ) [sec]	1.957	0.838	0.838	0.699

Table S5. Detailed NMR parameters of compound **5**.

spectra	^{1}H	^{13}C	DEPT90	DEPT135
pulse sequence	zg30	zgdc	dept90	dept135
temperature [k]	300.0	300.0	300.0	300.0
relaxation delay (D1) time [sec]	1	2	2	2
number of scans (NS)	4	256	64	128
frequency [MHz]	600	150	150	150
sweep width (SW) [Hz]	16741	39062	46875	46875
acquisition time (AQ) [sec]	1.957	0.838	0.699	0.699

Table S6. Detailed NMR parameters of compound **6**.

spectra	^{1}H	^{13}C	DEPT90	DEPT135
pulse sequence	zg	zgdc	dept90	dept135
temperature [k]	300.0	300.0	300.0	300.0
relaxation delay (D1) time [sec]	2	3	2	2
number of scans (NS)	4	148	29	96
frequency [MHz]	400	100	100	100
sweep width (SW) [Hz]	8012	24038	24038	23809
acquisition time (AQ) [sec]	4.089	1.363	1.363	1.376

Table S7. Detailed NMR parameters of compound **7**.

spectra	^{1}H	^{13}C	DEPT90	DEPT135
pulse sequence	zg30	zgdc	deptsp90	deptsp135
temperature [k]	300.0	300.0	300.0	300.0
relaxation delay (D1) time [sec]	10	2	2	2
number of scans (NS)	4	512	256	256
frequency [MHz]	600	150	150	150
sweep width (SW) [Hz]	12019	36231	36231	36231
acquisition time (AQ) [sec]	2.726	0.904	0.904	0.904

Table S8. Detailed NMR parameters of compound **8**.
